# Examining nursing processes in primary care settings using the Chronic Care Model: an umbrella review

**DOI:** 10.1186/s12875-023-02089-3

**Published:** 2023-09-04

**Authors:** Emilie Dufour, Jolianne Bolduc, Jérôme Leclerc-Loiselle, Martin Charette, Isabelle Dufour, Denis Roy, Andrée-Anne Poirier, Arnaud Duhoux

**Affiliations:** 1 Faculty of Nursing, Université de Montréal, Montréal, Canada; 2 École de santé publique, Université de Montréal, Montréal, Canada; 3 School of Nursing, Université de Sherbrooke, Sherbrooke, Canada; 4 Department of Epidemiology, Biostatistics, and Occupational Health, McGill University, Montréal, Canada; 5 Commissaire à la santé et au bien-être, Gouvernement du Québec, Montréal, Canada; 6 Faculty of Medicine, Université Laval, Québec, Canada

**Keywords:** Chronic care model, Systematic review, Community Care, Nurse-led care, Chronic disease

## Abstract

**Background:**

While there is clear evidence that nurses can play a significant role in responding to the needs of populations with chronic conditions, there is a lack of consistency between and within primary care settings in the implementation of nursing processes for chronic disease management. Previous reviews have focused either on a specific model of care, populations with a single health condition, or a specific type of nurses. Since primary care nurses are involved in a wide range of services, a comprehensive perspective of effective nursing processes across primary care settings and chronic health conditions could allow for a better understanding of how to support them in a broader way across the primary care continuum. This systematic overview aims to provide a picture of the nursing processes and their characteristics in chronic disease management as reported in empirical studies, using the Chronic Care Model (CCM) conceptual approach.

**Methods:**

We conducted an umbrella review of systematic reviews published between 2005 and 2021 based on the recommendations of the Joanna Briggs Institute. The methodological quality was assessed independently by two reviewers using the AMSTAR 2 tool.

**Results:**

Twenty-six systematic reviews and meta-analyses were included, covering 394 primary studies. The methodological quality of most reviews was moderate. Self-care support processes show the most consistent positive outcomes across different conditions and primary care settings. Case management and nurse-led care show inconsistent outcomes. Most reviews report on the clinical components of the Chronic Care Model, with little mention of the decision support and clinical information systems components.

**Conclusions:**

Placing greater emphasis on decision support and clinical information systems could improve the implementation of nursing processes. While the need for an interdisciplinary approach to primary care is widely promoted, it is important that this approach not be viewed solely from a clinical perspective. The organization of care and resources need to be designed to support contributions from all providers to optimize the full range of services available to patients with chronic conditions.

**PROSPERO registration:**

CRD42021220004.

**Supplementary Information:**

The online version contains supplementary material available at 10.1186/s12875-023-02089-3.

## Introduction

The constant rise of chronic diseases and multimorbidity requires healthcare systems to consider ways to strengthen the performance of primary care [1–3]. There is clear evidence that nurses can play a significant role in responding to the needs of populations with chronic conditions, notably in providing effective lifestyle interventions and a patient-centered approach [4, 5]. A number of recent studies [6, 7] have highlighted an important lack of consistency both between and within primary care settings in the implementation of nursing processes for chronic disease management. Despite an increasing focus on the need to support interdisciplinary practice [8] the integration of a comprehensive range of providers is still a major challenge [9, 10]. Models of primary care remain heavily focused on medical care [10, 11] and tend to center around the needs of medical practice rather than the interprofessional team [9]. A full contribution from other providers is needed for high-performing primary care that is responsive to the full range of population needs [8]. These other contributions are used in widely varying ways in the predominant models of care [10].

Among these providers, nurses are the most represented group across the different primary care models [12–14]. They are also the group whose practice characteristics are most dependent on the models of care in which they work [15, 16]. Nurses are involved in several dimensions of the primary care service continuum, including home care, community ambulatory clinics, and family practice clinics [12, 17]. Their degree of autonomy, activities, and scope of practice depend largely on the organizational structures and work practices in place [6, 12]. Their contributions to primary care remain suboptimal in many ways [6, 16].

On one hand, other providers’ perceptions of nurses’ role in primary care contribute to an underutilization of their expertise [6, 18–20]. The studies by Al Sayah, Szafran [18] and Lukewich, Edge [6] report that other providers have a blurred view of nurses’ contribution to patient care. Unlike other providers in primary care, such as social workers or pharmacisst, nurses’ scope of practice is often perceived as undefined [21]. The range of nurses’ education levels, which modifies their scope of practice, is another factor that blurs the understanding of the role they play in primary care [16, 22].

On the other hand, nurses face a considerable challenge in taking ownership of their role in primary care. Primary care nursing practice is significantly different from that of a hospital setting, which is largely predominant across curricula [18]. Primary care requires nurses to work in a broad scope of practice with less defined parameters, within which they must display initiative and creativity [18]. The high degree of autonomy and independence that typically prevails in primary care can be challenging for some nurses [16]. For example, primary care typically involves a case management approach, while many nurses report working primarily from a task-specific perspective [18].

Ambiguous understanding of nurses’ contributions by other providers, together with nurses’ own difficulty in taking ownership of their scope of practice, contributes to fragmentation and duplication of care [16] and leaves nurses in an underutilized [14] and in some cases invisible role [21]. This creates challenges in capturing the impact of their practice on care and service outcomes, and further adds to the challenge of securing appropriate resources and organizational support for the full implementation of their activities [16].

Several reviews have been conducted on nursing activities in the management of chronic care diseases. Their findings focus either on a specific setting [23], populations with specific health conditions [24, 25], or a specific type of nurses [26, 27]. While being highly valuable in understanding the contributions of nursing, these findings focus on either a clinical or an organizational perspective. Based on the Chronic Care Model (CCM), this review examines the processes of nurses in primary care using a comprehensive perspective. The CCM is halfway between the clinical and organizational dimensions of effective care in the management of chronic conditions. As it describes both components of clinical processes, i.e. support for self-care, case management, and components in the organization that work to support providers in effectively performing their clinical processes, we suggest that analyzing nursing processes based on this framework will allow for mapping out tools, interventions and other levers that contribute to the effectiveness of nursing. In this overview we will be using the concept of nursing processes to capture both the clinical and organizational aspects of care delivery [28].

### Chronic care model

The CCM has been widely used over the few past decades to identify the components of effective interactions between patients with chronic conditions and primary care providers [29, 30]. This framework emphasizes attributes of responsive care for patients with long-term conditions in a healthcare system primarily oriented toward acute care. The CCM addresses (1) the interactions that take place in the clinical process to support the patient’s active participation in his self-care, and (2) how the provider needs to be supported in order to carry out these interactions in an optimal way. Support for patients and providers is addressed based on four main components in the CCM.

First, the CCM emphasizes that primary care should be organized as to support self-management that is focused on increasing patient’s and families’ skills so that they can effectively manage their conditions. Second, care and services should be designed as to optimize the allocation of tasks within teams and as to offer follow-up that meets the specific needs of patients with chronic conditions. Optimal follow-up may be achieved through flexibility in the organization of appointments and by facilitating follow-up as to patients’ needs change. The ability of professionals to provide this type of follow-up depends on strong continuity and coordination mechanisms being in place. Third, the CCM emphasizes the need for providers to be supported as to offer care based on best practices. This is achieved through use of guidelines, advanced education, access to training, use of decisions support tools and increased interactions with specialists. Fourth, clinical information systems should be an integral part of primary care. Information systems may be used to facilitate care planning, information transfer and to provide reminders to professionals, as well as a source of ongoing feedback on their performance to support their capacity to improve their practice.

### Objectives

This umbrella review builds on the CCM to provide a descriptive picture of primary care nursing processes reported in systematic reviews with a focus on how the practice is described, and the components in the organization that are used to support its implementation.

Specifically, we aimed to describe:

(1) Nursing processes that are associated with better outcomes.

(2) The characteristics of the nursing processes.

(3) The components of the Chronic Care Model covered in nursing processes.

## Methods

In order to determine patterns among nursing processes and potential ways to support their implementation, we focused on studies that quantitatively assessed processes. A preliminary search showed a large number of quantitative primary studies covering the topic of chronic disease management by nurses in primary care. We therefore decided to limit this review to descriptive systematic reviews and meta-analyses. A protocol for the method was designed prior to the start of this overview and is available in the PROSPERO registry (CRD42021220004). The methodology is based on Joanna Briggs Institute [31] recommendations for umbrella reviews. Reporting was made in accordance with the *Preferred Reporting Items for Systematic Reviews and Meta-Analyses (PRISMA) Checklist* [32]. The checklist is available in Additional File 1.

### Eligibility criteria

Reviews were eligible if they satisfied the following criteria:

- Full-text systematic reviews published between 2005 and 2021 in English or French language.
- Searched at least 2 databases and conducted a risk of bias assessment.
- Systematic reviews of quantitative primary studies (systematic reviews including both quantitative and qualitative data were included only if data were analysed separately).
- Primary care must be the primary setting (including community, home care, general practice).
- Focus on a nursing process: any activity, care, service, intervention performed or that characterizes the provision of care by a primary care nurse.
- For reviews including other healthcare providers nurses must represent 80% or more of the providers or the data must be analysed separately.
- Focus on an adult population with at least one chronic condition.
- Report an outcome related to the patient’s condition (physical, psychological, cognitive) or to the delivery of care and services.

Reviews were excluded if any of the following were present:

- Focus on palliative care or end-of-life care.
- Focus on long-term facilities.
- Focus only on a care facility (ex: nurse-led clinic with no description of nurse-led processes).
- Focus on the effects of medication or a technique.
- Qualitative evidence syntheses.
- Non-systematic reviews.

### Search strategy

An information specialist was involved in the research strategy development. A comprehensive search strategy was used to identify reviews. Four databases were searched in January 2021: MEDLINE (Ovid), EMBASE (Ovid), Cumulative Index for Nursing and Allied Health Sciences (CINAHL) and EBM Reviews *(including Cochrane Database of Systematic Reviews; Database of Abstracts of Reviews of Effects; Health Technology Assessment; NHS Economic Evaluation Databa*se). A sample search strategy is available in Additional File 2. The Centre for Reviews and Dissemination systematic reviews search filters were applied to the search strategy as suggested by Joanna Briggs Institute [31]. Grey literature was searched using the Grey Matters tool designed by the Canadian Agency for Drugs and Technologies in Health (CADTH). Reference lists of the included papers were reviewed. Prospero register was consulted to identify any additional review.

### Study selection

First, all literature to screen was imported into Endnote [33] to facilitate organization and removal of duplicates. We used Covidence© [34] an online citation screening tool, to facilitate and monitor study selection. Two reviewers independently reviewed abstracts and titles. Full-text articles of included abstracts were retrieved and reviewed for inclusion by two independent reviewers. Any discrepancies were resolved through discussion. A third reviewer was consulted when consensus was not reached.

### Data extraction

Data extraction was performed by two independent reviewers with a standardized form. This form was pre-tested in a pilot phase and did not require any subsequent modifications. Differences in data extraction were discussed with a third reviewer if necessary. The standardized form summarized the following information: objectives, participants and numbers, funding, primary studies included and study designs. Data on nursing practice were extracted by outcome measure. Information on the activity and process characteristics, (including setting, frequency, length, mode of delivery, any tool or strategy used), usual care and study follow-up was extracted for each outcome measure.

### Methodological quality appraisal

Two reviewers independently assessed the methodological quality of the reviews using the AMSTAR 2 tool [35]. Assessment was performed using a form based of AMSTAR 2 domains and transferred into the Covidence software. The reviewers assigned a score for each of the 16 domains. Differences between reviewers were resolved after discussion when necessary. As suggested by Shea et al. [35], the overall assessment was primarily based on seven critical domainss. We placed specific emphasis on ratings of domain 13, which relates to authors’ consideration of the risk of bias in primary studies in the synthesis of systematic reviews, and domains 11 and 14 on the appropriateness of statistical analyses. As a result of this assessment, we determined overall scores ranging from high to very low confidence in the results.

### Data synthesis

Results were synthesized and tabulated with a narrative summary. Results were grouped according to nursing processes and outcome measures. Given the high heterogeneity observed between studies in terms of interventions, populations, and outcomes, no additional statistical analysis was performed.

## Results

### Results of the search process

**Fig. 1:**
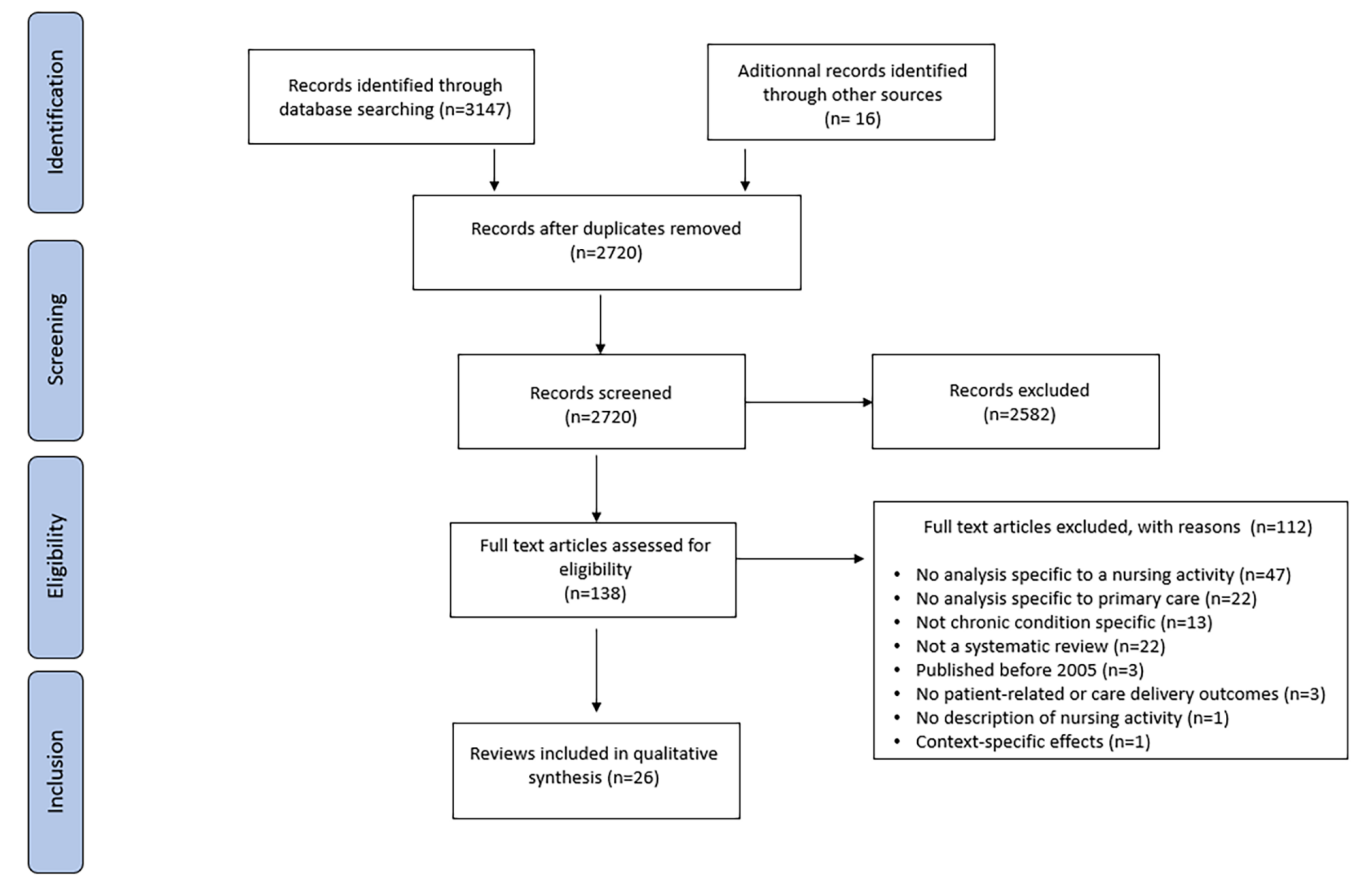
PRISMA flow diagram of search results

Twenty-six systematic reviews were included. The Preferred Reporting Items for Systematic Reviews and Meta-Analyses (PRISMA) [32] flow chart of the study selection is depicted in Fig. 1. The list of excluded full-texts and the reasons for their exclusion are provided in Additional File 3.

### Description of included systematic reviews

The publication dates of the primary studies ranged from 1967 to 2019, with nearly all published after 2000. The vast majority of the 394 primary studies were randomised controlled trials (RCTs). Other study designs included non-randomised controlled trials (NRCTs), controlled before and after intervention studies and observational studies. Forty-three primary studies were in overlap in more than one systematic review, the majority of which related to diabetic populations and Hemoglobin A1C (HbA1c) outcome measures. Two reviews [36] examining the prescribing activity on diabetic patients included the same seven studies. This was the only case where the overlap involved the same population, intervention, and outcome. A summary of the included reviews and their methodological quality assessment is reported in Table 1.

**Table 1:** Summary of included systematic reviews and reporting of methodological quality assessment

Author and year, type of synthesis	Study period	Number of included studies and designs	Number of participants	Population and health condition	Main nursing process	Overall confidence in the results based on AMSTAR 2
Backhouse et al. (2017) Meta-analysis	1999–2014	14 RCTs	10,372	Adults with a dementia diagnosis of any type living at home	Case management	Moderate
Baker et Fatoye (2017) Descriptive	2004–2016	26 articles describing 20 RCTs	3384	Adults with COPD	Self-management program	Low-Moderate
Caro-Bautista et al. (2020) Meta-analysis	2005–2016	21 articles describing 20 RCTs	12,018	Adults with type 2 diabetes	Self-care programmes	High
Clark et al. (2010) Meta-analysis	1979–2008	33 RCTs	19,069	Adults aged 18 or over with hypertension	Nurse-led care Community monitoring	High
Crowe et al. (2019) Descriptive	2003–2018	18 studies: 10 RCTs 3 Open Label Designs 2 Qualitatives 1 historical control 1 audit 1 retrospective cohort study	33,971	Adults witih type 2 diabetes	Nurse-led interventions	Low - Moderate
Deschodt et al. (2020) Meta-analysis	2004–2018	21 articles reporting 19 studies: 5 Cluster-RCT 4 RCTs 3 NR controlled trial 1 BA 2 Controlled BA 1 Cluster RCT 1 Pseudo cluster-randomized controlled trial 2 Cluster NR controlled trial	22,168	Adults aged ≥ 65 or a reported mean age of ≥ 75 years	Nurse-led services	Moderate
Dhar et al. (2020) Descriptive	2009–2018	1 Quantitative judgement analysis 1 Quasi experimental 1 Pre/ postintervention 1 Nonrandomized controlled trial 1 Randomised controlled trial 1 Retrospective audit 2 Descriptive 1 Quantitative cost analysis 1 Comparative	1062	Adults with a chronic wound	Nurse-led care	Critically low
Ekers et al. (2013) Meta-analysis	2003–2013	14 RCTs	4440	Adults with a primary diagnosis of depression and one or more long-term physical health problems	Case management	High
Facchinetti et al. (2020) Meta-analysis	1994–2018	30 RCTs	8920	65 years and over patients with one or more chronic diseases who were discharged home from hospital	Continuity of care	High
Gorina et al. (2018) Descriptive	2004–2015	20 RCTs	15,439	Adults with type 2 diabetes, hypertension and hypercholoesterolemia	Educational interventions	Moderate-High
Halcomb et al. (2019) Descriptive	1998–2016	9 RCTs	2404	Adults with a diagnosis of mental illness	Case management	Moderate
Han et al. (2019) Descriptive	1991–2016	28 studies: 12 RCTs 5 NR controlled studies 7 Single group BA 1 Cross-sectional study 1 Qualitative 1 Mixed-methods	27,055	Community-dwelling individuals with CVD and/or CVD risk factors	Nurse-led services	Moderate
Huntley et al. (2016) Meta-analysis	1993–2012	32 articles reporting 22 studies: 17 RCTs 5 NRCTs	8626	Adults with heart failure	Case management	Moderate
Latour et al. (2007) Descriptive		10 studies: 8 RCTs 2 controlled before/ after studies	5092	Patients over 18 years of age and were defined as complex; patients with acute or chronic medical condition(s) and described other vulnerabilities	Case management	Moderate
Massimi et al. (2017) Meta-analysis	2000–2013	29 articles reporting 23 RCTs	10,162	Patients > 18 years old with a diagnosis of chronic disease or multiple morbidity	Educational intervention	High
Oeseburg et al. (2009) Descriptive	1997–2004	8 RCTs	14,587	Frail older people	Case management intervention	Moderate
Osakwe et al. (2020) Descriptive	1995–2018	7 studies: 2 RCTs 3 Quasi-experimental 1 Observational cohort study 1 Mixed-methods program evaluation	1748	Adults ≥ 65 years old post hospital discharge	Nurse-led services	Low - Moderate
Parker et al. (2016) Meta-analysis	2003–2013	7 studies: 2 Cluster RCTs 2 RCTs 1 Cluster observational cohort study 1 Cluster controlled BA study	18,379	Adult participants (aged above 18 years) with T2D	Nurse-led services	Moderate
Rice et al. (2018) Descriptive	1967–2010	7 RCTs	3549	Adults 18 years and older with HF	Nurse-led education	Low - Moderate
Schadewaldt et Schultz (2011) Meta-analysis	1998–2007	13 articles reporting 7 RCTs	3246	Adults (aged > 18 years) admitted to a hospital or a general practice with newly diagnosed or existing coronary heart disease	Nurse-led care	Low
Tabesh et al. (2018) Descriptive	2002–2011	9 RCTs	1974	Adults aged 18 years with T2D	Nurse prescribing	Moderate
Taylor et al. (2005) Meta-analysis	1987–2003	9 RCTs	1057	Patients with COPD	Case management	Low
Vermeire et al. (2005) Meta-analysis		21 studies: 14 RCTs 4 controlled BA 1 epidemiological study 1 cross-over study 1 controlled trial	4135	Participants with type 2 diabetes	Interventions for improving the adherence to treatment recommendations	Moderate
Wang et al. (2019) Meta-analysis	1998–2015	17 RCTs	2701	Adults with type 2 diabetes	Nurse prescribing	Critically low
Wong et al. (2012) Meta-analysis	1987–2006	9 RCTs	1498	Participants with COPD	Nurse-led care	Moderate
Yu-Mei Chen et al. (2019) Meta-analysis	1995–2016	12 RCTs	3030	Adults (age 18 & above) with T2D	Nurse-led tele-coaching	Low

BA, before-after; COPD, Chronic Obstructive Pulmonary Disease; CVD, Cardiovascular disease; HF, Heart failure; RCT, Randomized Controlled Trial; T2D, Type 2 diabetes

### Methodological quality

An overall high confidence score was assigned to six reviews, a moderate score was assigned to 15 reviews, a low score to three , and a critically low score to two. No review was excluded from the analysis based on quality assessment, but quality was considered in the results and their interpretation. The main problematic areas for most reviews pertained to the literature search strategy and the registration of a protocol prior to conducting the reviews. Most of the reviews did not provide a list of excluded articles nor justification for their exclusion. Several reviews were rated as having a partial comprehensive literature research mainly because justifications for restrictions were not provided. Almost all included meta-analyses used adequate methods for combining of results. The detailed ratings for each domain of AMSTAR 2 are reported in Additional File 4.

### Description of nursing processes and outcomes

Nursing processes described across the 26 reviews were classified in five broad categories. A description of the processes based on the information reported in the reviews are presented in Table 2 and a summary of the effectiveness of the five nursing processes is reported in Table 3.

**Table 2:** Description of nursing processes

Nursing processes		Description
Activities	Case management	Characterized by an emphasis on assessment of signs and symptoms, medications, lifestyle, environment, and care planning and coordination. Often involves referral and promotion of options and services. May include education that is not the primary component.
	Self-management support	Characterized by a structured delivery of education. Frequently involves strategies such as motivational interviewing, goal setting, action planning.
	Prescribing	Characterized by care where the nurse is responsible for determining and/or adjusting the dosage of a treatment, medication.
Delivery models	Continuity	Characterized by a focus on one or more of the three continuity dimensions. Relational continuity refers to interpersonal relationships between the patient and the provider, informational continuity is related to the availability of documentation and transfer of information between settings, and management continuity focuses on offering flexible care that responds to patients’ changing health status and needs.
	Nurse-led care	Characterized by central involvement of nurses in the monitoring, treatment planning and assessment of chronic conditions. Includes more than one form of intervention. Most of these practices are compared to those of general practitioners.

**Table 3:** Findings of the overall impact of five nursing processes on reported outcomes

Nursing process	First author of review	Magnitude of positive effects	Overall risk of bias score as reported by authors of reviews
Case management	Backhouse	• **Caregiver quality of life** - interventions using a nurse case manager vs. other provider: SMD = 0.94 vs. 0.03 respectively; p < 0.001	Moderate to high
	Ekers	• **Depression symptom level**: Moderate improvement d = 0.43; 95% CI 0.34 - 0.52 p < 0.001 heterogeneity I^2^ = 36.68%	Low-moderate
	Halcomb	• **Depression symptoms**: small to moderate improvements in 5 out of 8 studies • **Functional outcomes**: small and inconsistent significant improvements in 3 out of 4 studies	N/A
	Huntley	• **Length of stay**: MD = − 1.28 days; 95% CI − 2.04 to − 0.52 p = 0.001; heterogeneity I2 = 63% • **Hospital readmission**: Rate ratio = 0.74; 95% CI 0.60 to 0.9 p = 0.008; heterogeneity I^2^ = 69%	Low (5) High or unknown (5)
	Latour	• **Hospital days**: small and inconsistent improvements	Low (6) Moderate-high (4)
	Oeseburg	None.	Low (2) Moderate (4) High (2)
	Taylor	None.	Moderate-high (3) High (6)
Self-management support	Baker	• **Anxiety**: significant improvement in 3 out of 5 studies – no effect size reported in primary studies • **Self-efficacy**: significant improvements in 6 out of 10 studies – no effect size reported in primary studies • **Physician visits**: significant differences reported in 3 out of 5 studies – no effect size reported in primary studies	Moderate
	Caro-Bautista	• **HbA1c** : Programmes in which nurses participated in the intervention vs. programmes without nurses participating **Short-term**: MD − 0.32% 95% CI: −0.57% to − 0.07% versus. −0.25%; 95% CI: −0.60–0.11% **Mid-term**: MD − 0.38%; 95% CI: −0.74% to − 0.02% versus. −0.20%; 95% CI: −0.54–0.13%	Moderate-high
	Gorina	• **Systolic and diastolic blood pressure**: moderate significant improvements in 8 out of 12 studies • **Changes in physical activity practice**: moderate significant improvements in 2 out of 2 studies • **Total cholesterol, high-density cholesterol, low-density cholesterol, and triglyceride**: small significant improvements in 5 out of 8 studies • **Change in nutritional habits**: small improvement in 1 single study	Low (1) Uncertain (14) High (5)
	Massimi	• **HbA1c**: MD -0.15, 95% CI -0.32 to 0.01, heterogeneity I2 = 28%, p = 0.21 • **SBP**: MD -3.04, 95% CI -5.01 to -1.06, heterogeneity I2 = 55%, p = 0.02 • **DBP**: MD -1.42, 95% CI -2.36 to -0.49, heterogeneity I2 = 34%, p = 0.14	Low (9) Moderate (9) High (2)
	Rice	• **Readmission**: small significant improvements in 3 out of 3 studies – no effect size reported • **Hospitalization**: small significant improvements in 2 out of 3 studies – no effect size reported	N/A
	Vermeire	• **HbA1c**: minimal effects in 2 out of 2 studies MD -0.10, 95% CI -0.12 to -0.08, p < 0.00001; heterogeneity: 0.0%	Low (3) Moderate (13) High (5)
	Yu-Mei Chen	• **SBP**: MD -2.22, 95%CI -3.59 to -0.49, heterogeneity: I2 = 0%, p = 0.55	N/A
Prescribing	Tabesh	None.	Moderate
	Wang	None.	Low (8) High (9)
Nurse-led care	Clark	*Community monitoring*: • **SBP**: MD -4.8 mmHg, 95% CI − 7.0 to − 2.7, heterogeneity: I²=0%, p = 0.51 • **DBP**: MD − 3.5, 95% CI − 4.5 to − 2.5, heterogeneity: I²=0%, p = 0.54 *Nursing follow-up*: • **SBP**: MD -3.48, 95% CI -5.88 to -1.08, heterogeneity: I² = 36%, p = 0.16 • **DBP**:MD -1.92, 95% CI -3.39 to -0.45, heterogeneity: I²= 43%, p = 0.12	Moderate
	Crowe	• **HbA1c**: small to moderate significant improvements in 9 out of 15 studies • **Blood pressure**: small significant improvements in 5 out of 7 studies • **Patient satisfaction**: improvements in 2 out of 2 studies – no effect size reported	Low-moderate
	Deschodt	None.	Considerable
	Dhar	• **Pain**: small improvements in 4 out of 4 studies – no effect size reported • **Wound size**: moderate improvements in 2 out for 3 studies – no effect size reported • **Economic outcomes**: small improvement in 1 study	Moderate-low
	Han	• **Self-care**: moderate significant improvement in 9 out of 9 studies • **Quality of life**: moderate significant improvement in 2 out of 2 studies • **Healthcare outcomes**: moderate significant improvement in 11 out of 11 studies • **Healthcare use of services**: moderate significant improvements in 8 out of 8 studies	Moderate
	Osakwe	• **ED visits**: moderate significant improvement in 2 out of 3 studies • **Quality of life**: moderate significant improvement in 1 single study	Low (1) Uncertain (2) High (4)
	Parker	• **SBP**: MD -4.40, 95%CI -7.06 to -1.74, heterogeneity: I^2^ = 59%, p = 0.06 • **DBP**: MD -2.96 95%CI -4.97 to -0.96, heterogeneity: I^2^ = 78% p = 0.004	Acceptable
	Schadewaldt	• **Quality of life**: small and inconsistent improvements in 4 out of 4 studies	N/A
	Wong	• **Health related quality of life – disease specific**: MD -2.60, 95% CI -4.81 to -0.39, heterogeneity: I²= 0% p = 0.77 • **General quality of life**: small and inconsistent improvements in 2 out of 3 studies	Moderate-high
Continuity	Facchinetti	• **Readmission rate at one month**: RR 0.84, 95% CI 0.71 to 0.99, heterogeneity I^2^ = 3% p = 0.41 • **Readmission rates at 1–3 months**: RR 0.74, 95% 0.65 to 0.84, heterogeneity I^2^ = 7% p = 0.38 • **Readmission rates at 6 to 12 months**: RR 0.84, 95% CI 0.74 to 0.95, heterogeneity: I^2^ = 51% p = 0.02	Low-moderate

### Case management

Case management was the main component of interventions reported in seven reviews [37–43]. These reviews included 79 RCTs, five NRCTs and two controlled before/after (BA) studies. All seven reviews were rated as being of moderate or high quality based on AMSTAR 2 domains. Most of the primary studies reported in these reviews had a moderate risk of bias.

Five of the reviews reported significant differences for at least one outcome measure. Only two of the five reviews that included outcomes on healthcare service use (including hospitalization, emergency department visits, nursing home admissions, hospital (re)admission, length of hospital stay) reported significant differences in favor of nurse-led case management. Huntley, Johnson [38] reported significant decreases in length of stay related to community and hospital-initiated case management and a significant decrease in hospital readmission in favor of hospital-initiated case management for patients with heart failure. The authors considered the primary studies included in this meta-analysis to have an overall low to moderate risk of bias. Latour, van der Windt [39] reported significant in hospital days following nurse-led case management for patients with a complex condition. Five of the six studies addressing this outcome were considered to be at low risk of bias by the authors.

Ekers, Murphy [41] focused on case management targeting patients with a diagnosis of depression. This meta-analysis reported a moderate improvement in depression symptom level at follow up (*d* = 0.43 95% CI 0.34 to 0.52 p > 0.001 heterogeneity I^2^ = 36.68%). The authors reported a low-moderate overall risk of bias for the primary studies. Another systematic review addressing case management for people with mental illness [37] also reported significant improvements in depression symptoms in five of its eight primary studies addressing this outcome. The authors of this review gave to the studies an overall low risk of bias score.

A meta-analysis targeting older adults with dementia [42] reported minimal improvements for case management by nurses. The only significant impact was related to caregiver quality of life, while outcomes of hospitalization, admission to nursing homes and mortality showed no significant improvement. Overall score for risk of bias of the included studies was moderate. Another review focussing on older adults [40] found no significant improvement in all outcomes related to health service use and costs.

Finally, a meta-analysis targeting COPD patients [43] found no significant impact for nurse-led case management in the reported twelve reported outcomes. Six of the nine included primary studies were considered at high risk of bias by the authors.

### Self-management support

Self-management support was the main component of interventions reported in seven reviews [24, 44–49]. These reviews included 105 RCTs, four controlled before and after studies, one epidemiological study, one cross-sectional study and one controlled trial. All seven reviews reported significant differences for at least one outcome measure.

Five of the systematic reviews involved patients with type 2 diabetes [44–46, 48, 49]. Educational interventions showed significant improvements of HbA1c in three of these reviews. Educational programmes provided by nurses achieved better results compared to those with no nurses participating at short and mid terms in one meta-analysis [44]. The authors reported an overall moderate-high score for risk of bias and mentioned that studies with very low methodological quality were eliminated from the review. Massimi, De Vito [46] reported a positive trend for nurse-led self-management support interventions on HbA1c in a meta-analysis including 10 studies (Mean difference (MD) -0.15, 95% CI -0.32 to 0.01, heterogeneity I^2^ = 28%, p = 0.21). Authors considered three included studies to be of low quality, two of moderate quality and two of high quality [46]. Vermeire, Wens [48] reported significant improvements in the two studies addressing HbA1c following nurse-led interventions for improving adherence to treatment recommendations. Most of the studies included in this review were assessed as having a moderate risk of bias. Yu-Mei et al.’s review [49] on effects of tele-coaching showed minimal improvement on HbA1c, as did the educational interventions reported in Gorina, Limonero [45].

A systematic review addressing the effectiveness of nurse-led self-management for COPD patients [24] reported improvements related to anxiety symptoms and self-efficacy in most of the studies addressing those outcomes. Interventions were also associated with a significant difference in physician visits in most of the included studies. However, all other outcome measures, including quality of life, emergency department (ED) visits, hospital admission and costs showed no significant improvement in the majority of included primary studies. The authors considered the studies to have an overall low to moderate risk of bias.

Four reviews reported blood pressure outcome measures [44–46, 49], of which three reported positive impact of self-management support. Caro-Bautista, Kaknani-Uttumchandani [44] reported that studies involving a nurse as main provider were associated with a significant decrease in systolic blood pressure (SBP). Eight of the twelve primary studies included in Gorina et al.’s systematic review (2018) showed significant decrease in blood pressure in favor of the intervention group following educational interventions led by nurses. A meta-analysis addressing self-management support interventions targeting various chronic diseases [46] showed a significant improvement both in systolic and diastolic blood pressure based on ten studies (MD -3.04, 95%CI -5.01 to -1.06, heterogeneity I^2^ = 55%, p = 0.02 and MD -1.42, 95% CI -2.36 to -0.49, heterogeneity I^2^ = 34% (p = 0.14). Yu-Mei Chen et al.’s meta-analysis (2019) also showed overall significant impact in favor of the intervention on SBP based on 4 studies (MD -2.22, 95%CI -3.59 to -0.49, heterogeneity: I^2^ = 0%, p = 0.55. These authors did not report an overall score for the risk of bias of included studies. Finally, Rice, Say [47] reported significant impact of nurse-led education for patients with heart failure on both readmission and hospitalization in a majority of the studies reporting on those outcomes. No positive impact was demonstrated in any of the studies addressing quality of life and costs. The overall risk of bias for the included studies was considered as low by the authors.

### Prescription

Nurse prescribing was the main intervention reported in two reviews [36, 50] comprising 26 RCTs, that were rated as moderate and critically low quality, respectively. Most studies included in the reviews did not show significant impact of nurse prescribing. HbA1c was the only outcome measured at follow-up in both reviews. Tabesh, Magliano [36] reported a small difference in favor of the intervention group with the pooling of five studies reporting nurse prescribing as part of a team rather than independently (MD -0.34, 95%CI -0.71 to 0.02, heterogeneity I^2^ = 60%, p = 0.04). Nurses were reported to have followed algorithms or protocols for prescribing medications [36].

### Continuity

The effectiveness for continuity of care was reported in a single review [51] that included 30 RCTs with a large majority of nurses (83% of included studies) and some allied health professionals. This review obtained a high-quality rating based on AMSTAR 2 tool. Facchinetti, D’Angelo [51] focused their meta-analysis on the impact of relational, informational and management continuity in the care of older people with chronic illness(es) on readmission rates. Continuity was associated with lower readmission rate at one month with pooling of 10 studies (RR 0.84, 95% CI 0.71 to 0.99, heterogeneity I^2^ = 3% p = 0.41). Significant lower readmission rates at 1–3 months were also observed with the pooling of 11 studies (RR 0.74, 95% 0.65 to 0.84, heterogeneity I^2^ = 7% p = 0.38). Results for long term admission rates were equivocal. Readmission rates at 3 and 6 months did not decrease significantly. Readmission rates at 6 to 12 months were significantly lower, however pooling of the 13 studies showed significant heterogeneity (RR 0.84, 95% CI 0.74 to 0.95, heterogeneity: I^2^ = 51%). Subgroup analyses showed that studies that addressed all three dimensions of continuity, namely relational, informational and management continuity, were associated with a significant effect at short and long term. The authors concluded that continuity may prevent short- term readmission for older people but that its effect on long-term readmission is inconclusive. The primary studies included in the review were considered as having an overall moderate risk of bias by the authors.

### Nurse-led care

The effectiveness of nurse-led care was reported in ten reviews [25, 52–59]. These reviews comprised 87 RCTs and 51 nonexperimental studies.

Reviews that focused on nurse-led care had AMSTAR 2 quality ratings ranging from low to moderate, with most being of moderate quality. A significant proportion of the included primary studies were assessed by the authors as having a moderate to high risk of bias. Two reviews reported the effects of a set of nurse-led interventions targeting patients with diabetes [25, 57]. Both reviews reported positive impact of the interventions on blood pressure. Parker, Maresco-Pennisi [25] reported significant decreases of systolic (MD -4.40, 95%CI -7.06 to -1.74, heterogeneity: I2 = 59% p = 0.06) and diastolic blood pressure (MD -2.96 95%CI -4.97 to -0.96, heterogeneity: I2 = 78% p = 0.004) at 12-month follow-up. Crowe, Jones [57] reported significant improved glycemic control at follow-up for seven of the eleven studies addressing this outcome, while Parker et al.’s meta-analysis [25] showed no significant difference for this outcome.

Two reviews examined nurse-led care for older adults [52, 58] in home care settings. The meta-analysis by Deschodt, Laurent [52] found no significant improvement in any of the six outcome measures, including quality of life, mortality, and use of healthcare services. The authors considered the included primary studies to be at considerable risk of bias. Osakwe, Aliyu [58] whose review focused on interventions delivered by nurse practitioners (NPs), reported a significant decrease in ED visits in two of the three studies addressing this outcome. The authors also reported a significant improvement in quality of life, although only one study addressed this outcome measure. Four of the included studies were considered at high risk of bias, while two were considered uncertain and one was considered at low risk of bias.

Han, Quek [53] and Schadewaldt and Schultz [54] both reported the effects of a nurse-led set of interventions targeting patients with cardiovascular disease. Han, Quek [53] reported significant impact in most included studies for all four outcome measures. The authors considered primary studies to be at moderate risk of bias. Reported outcomes included self-care, quality of life, mortality and healthcare use of services. The effects reported by Schadewaldt and Schultz [54] were not as favourable. This meta-analysis reported significant changes for one of the eleven outcome measures. Significant change was reported on quality of life. No change was achieved on blood pressure, total cholesterol, compliance nor hospital admission. An overall score for risk of bias was not reported in this review.

A meta-analysis on the management of hypertension [55] reported significant improvements in blood pressure for two types of nurse-led services. Community monitoring of hypertension demonstrated a significant change in systolic and diastolic blood pressure from baseline (MD -4.8 mmHg, 95% CI − 7.0 to − 2.7, heterogeneity: I²=0% p = 0.51) and (MD − 3.5, 95% CI − 4.5 to − 2.5, heterogeneity: I²=0% p = 0.54). Nursing follow-up in primary care nurse-led clinics achieved similar changes both in systolic and diastolic blood pressure (MD -3.48, 95% CI -5.88 to -1.08, heterogeneity: I² = 36% p = 0.16) and (MD -1.92, 95% CI -3.39 to -0.45, heterogeneity: I²= 43% p = 0.12). The authors considered the included primary studies to have an overall moderate risk of bias.

A meta-analysis by Wong, Carson [59] on nurse-led intervention in home care settings for COPD patients reported no significant change in the majority of 11 outcome measures. The only positive change was related to health-related quality of life (MD -2.60, 95% CI -4.81 to -0.39, heterogeneity: I²= 0% p = 0.77). Outcomes related to healthcare use of services, mortality and health status did not demonstrate significant change. One included study reported significant higher costs for intervention group.

Finally, the systematic review by Dhar, Needham [56] reported significant improvements in pain and wound size for adults with chronic wounds receiving nurse-led wound in community. Overall improvements were not significant for quality of life and rate of wound healing. Two studies included in this review reported economic outcomes. A cost analysis reported an annual savings of $28,341 USD on health care expenditures following the implementation of a wound contact nurse in the community. Wound care conducted by a trained nurse in the community demonstrated a saving of ₤4814 GBP per patient, compared with inpatient treatment. The authors of the review considered most of the included primary studies two be at moderate-low risk of bias. We rated this review as being at high risk of bias as it did not meet several criteria of the AMSTAR 2 grid.

### Characteristics of processes

#### Settings

The overview of all included reviews suggests that patients with chronic conditions received nursing care in a variety of settings. Most reviews reported studies in which nursing processes were performed in both home and ambulatory care settings, including general practice, outpatient clinic, primary care clinic, local community activity centers, nurse-led clinics and medical centers. Self-management support processes, which frequently included group sessions, were more likely to take place in outpatient settings [24, 44, 45], while case management was mainly conducted through home visits [40, 42].

#### Competencies of providers

Nurses’ competencies were addressed in the reviews through (1) education level and (2) specific training related to a practice or intervention. Although most studies reported on nurse training and education, only one meta-analysis including 23 RCTs [46] assessed the presence or absence of additional training for nurses providing self-management support interventions as an influencing factor. Stratified meta-analyses showed a significant impact of additional training received by nurses (MD -1.56, 95% CI 2.63 to 0.48) on blood pressure levels and HbA1c, whereas level of education did not influence the results for either outcome. Facchinetti, D’Angelo [51], whose meta-analysis reported significant associations between continuity of care on short-term readmissions of older adults, noted that most of the interventions were conducted by nurses with advanced competencies.

Three reviews reported initial or additional education as a factor influencing the effectiveness of nursing processes. Halcomb, McInnes [37] whom reported favourable impact of case management on depression symptoms and functional outcomes, included only general practices nurses who received specific training prior to the intervention. The majority of nurses involved in hypertension monitoring reported in Clark et al.’s meta-analysis [55] had either advanced competencies or received training and support from diabetes specialist nurses. Two studies reported the use of algorithms as a decision support tool. Analyses demonstrated an overall significant impact of the monitoring on blood pressure. Han, Quek [53] in part attributed the favorable effects of nursing care for patients with cardiovascular disease to the nurses’ high level of education or additional training received before the intervention.

Parker et al.’s meta-analysis [25] focused on the effectiveness of non-specialist nurses in the management of diabetic patients. Modest but significant changes were reported for most of the outcome measures, including blood pressure and HbA1c. The authors did not report on any further training received by the nurses prior to the intervention but noted that their interventions were supported by the use of algorithms in most studies. Authors also reported the access to a computer decision support system in two of the seven included studies.

We observed an overall inconsistency between reviews regarding the terminology used to describe the types of nurses. Very few studies clearly indicated the level of education associated with nursing credentials. This may limit the extent to which the influence of education level on outcomes can be determined compared to that of additional training.

### Chronic care model components reported in the systematic reviews

The results of the nursing processes in the management of chronic disease emphasize a concentration of processes on the system delivery design and self-management support components of the CCM. The decision support component is indirectly addressed by the regular mention of nurses’ use of guidelines to support their processes in primary studies, although it is not emphasized as a key feature of processes in most reviews.

Other decision support elements were addressed in a few reviews. This component of CCM encompasses education and training of providers, access to specialists and access to guidelines and algorithms. The five reviews that addressed specifically nurses’ competencies [25, 37, 46, 51, 53] all reported that interventions were performed by nurses with advanced degrees, who received specific training prior to the interventions, or who were supported by nurses and other specialized health providers. All of these reviews reported on the significant improvement in at least one of their primary outcome measures.

There was very little mention of the clinical information system component in any of the reviews either in the form of computerized tracking, system reminders, registries or feedback systems [60]. One review [52] with no overall significant effects for nurse-led care with home-dwelling older people reported a scarce use of technology and information systems, namely clinical administrative data, electronic medical records, and computerized algorithms. Oeseburg, Wynia [40] noted that the only study that showed significant improved outcomes associated with nurse case management was the only one that reported using technology to plan, organize and coordinate care.

## Discussion

This overview of systematic reviews summarised evidence on nursing processes and their characteristics for populations with chronic conditions in primary care settings.

### Summary of findings on nursing processes and related outcomes

Self-management support is the only activity that has been shown to be consistently effective across settings and populations. All seven reviews reported at least one improved outcome following self-management support delivery by nurses. The majority of reviews were assessed with the AMSTAR tool as being of moderate quality, while three of them were rated as high quality. Most of the primary studies reported in these reviews had a moderate risk of bias. Significant impact of additional training received by the nurses prior to the intervention was reported in one meta-analysis [46]. These findings are consistent with other literature on chronic disease management by nurses, which places great emphasis on their contribution in self-management support  [61, 62].

The effectiveness of nurse prescribing is inconclusive in this overview. Prescribing interventions have only been described in two reviews [36, 50] that targeted a single population, namely diabetic patients and that were rated as moderate and very low quality, respectively. The primary studies, several of which overlapped between the two reviews, were assessed as having an overall moderate risk of bias. Nursing prescribing was addressed in some of the other reviews indirectly, for example through mention of algorithms to support medication adjustment [25, 55]. However, because this activity was not the focus, it was not possible to assess its effectiveness from these data. The lack of reported positive effect for prescribing must therefore be interpreted considering the limitations in distinguishing its effects from those of other processes. The systematic review by Bhanbhro, Drennan [63] on the prescribing on nurses and other allied health professionals reported that very few studies had assessed the effectiveness of prescribing on health outcomes, highlighting how limited evidence was. The findings from this review are rather consistent with our own findings, which suggest that there is a need for high quality studies in nurse prescribing.

The effectiveness of the remaining processes was highly variable. The effectiveness of case management was inconsistent across all seven reviews, which is consistent with other reviews [64, 65]. Case management is often described as complex in that it is multifaceted and involves comprehensive assessment, coordination, and follow-up [66]. In this overview, it was the nursing process most often performed with populations presenting complexity factors, namely advanced age and comorbidities. All seven reviews were rated as being of moderate or high quality based on AMSTAR 2 domains. Most of the primary studies reported in these reviews had a moderate risk of bias. The inconsistency in the outcomes of case management interventions is partially aligned with the results of other systematic reviews, which report inconclusive effects on patient satisfaction and healthcare service use for people with multiple chronic conditions [3].

The review by Ekers et al. [41] reported outcomes of nurse-delivered collaborative care for depression and long-term physical conditions. This review which was assessed as being at low risk of bias and demonstrated the most consistent outcomes across primary care settings. This finding is consistent with a previous review on case management for patients with complexity factorss that reported improvement in depression symptoms [65]. Although no evidence was found on characteristics of delivery in terms of lengthand mode of delivery, nurses involved in most of the included studies had received additional training or had advanced competencies. Nurse-delivered collaborative care included proactive follow-up, monitoring of process and direct communication channels with specialist or primary care physician [41].

The effectiveness of nurse-led care was also inconsistent across reviews. Nurse-led care was particularly difficult to define since it combined several interventions delivered by nurses. Favourable and consistent outcomes were reported in four reviews [25, 53, 55, 56] three of which reported decision support and clinical information system use components. Nurse-led care for improving control of blood pressure in people with hypertension reported in Clark et al. [55] was provided by nurses with advanced training in most studies. Almost all the included studies also involved the use of protocols, algorithms or guidelines. The use of algorithms was also frequently mentioned in the review by Parker et al. [25], in support of management of type 2 diabetes by practice nurses. A few studies involved in this review also mentioned using recall systems.

A single review [51] reported results that focused on the continuity of care interventions by nurses, although continuity was also involved in case management processes. The review by Facchinetti et al. [51], which was considered to have a low risk of bias based on our AMSTAR 2 assessment, reported a significant overall impact of continuity on short-term readmission rates for older adults. The vast majority of interventions were reported to have been carried out by nurses with advanced competencies. A positive association was also found for studies involving the three dimensions of continuity, namely relational, informational and management continuity. This requires designing care so that encounters are performed by the same providers, information about previous events and conditions is used in care planning, and the plan is adapted as the patient’s needs change. The effects of nursing continuity have not been widely studied in other populations. In primary care, high levels of continuity of care have been associated with reductions in long-term mortality [66] and higher patient satisfaction [67].

### CCM components addressed in the reviews on nursing processes and implications for research and practice

In terms of the nature of nursing processes, this overview provides very similar findings to previous reviews that targeted a specific condition or setting, or category of nurses. This highlights that the role of nurses across the primary care continuum, for different chronic conditions and with different levels of training, revolves around the same clinical processes. Nursing processes that reflect the clinical components of the CCM, i.e., case management, self-care support, and monitoring, have been extensively reported in previous studies [6, 62, 68]. Reference to the support components of the CCM, which include clinical information systems and decision support, is virtually absent from all reviews. The most common aspect mentioned in support of nurses’ processes was nursing skills and training. Given that many of the strategies suggested by the CCM are currently not being addressed, we see greater emphasis and integration of all components as an opportunity to strengthen the role of nurses in primary care.

We found very little mention of the clinical information system component in the reviews. This suggests that information related to this component is not emphasized in the description of nursing processes or is not or only minimally integrated into nursing processes. Other reviews [69, 70] that have used the CCM to report on the effectiveness of primary programs for specific conditions also noted that the information system component was found among the interventions to a lesser extent, but tended to produce better outcomes.

A review of systematic reviews by Irwin, Stokes [71] reported positive effects for a number of practice-level quality improvement strategies that were based on the use of data from clinical information systems in primary care. These strategies included performance feedback and computerized reminders [71]. The use of computerized reminders with physicians has been associated with greater adherence to guidelines and better follow-up [69, 71]. Quality improvement collaboratives, which are based on performance feedback and problem-solving learning, have shown to support communication between providers and a better understanding and recognition of their respective roles within primary health care teams for the management of chronic care conditions [72]. Yet, there is no clear evidence of the extent to which nurses are able to actively participate in these strategies, considering that clinical information systems are still poorly used to provide data on nurses’ processes in primary care [73].

The CCM suggests that the use of data from clinical information system allows for a more concrete depiction of providers’ resource and service organization needs [60]. The nursing processes reported in this overview outline the priority areas of activity for which data could be gathered. Clinical information system could be used to support case management by providing feedback on the care trajectory and features of the caseload and to support continuity by providing information such as frequency of visits and follow-up with the same provider. Clinical information system could also provide data on the characteristics of the populations seen by nurses to tailor resources according to context-specific needs, for example, based on the prevalence of certain conditions or populations with complex needs. We recommend that future research focus on the review and evaluation of nursing-specific data-based feedback interventions.

The use of clinical information systems for computerized decision support has typically focused on improving prescribing practice with physicians [71, 74]. This strategy could be used with nurses to support their confidence in using their full scope of practice, including in prescribing, as well as in monitoring lab and blood pressure measurements [75]. A review on the use of clinical decision support with nurses [76] has previously highlighted the need for quantitative studies, as the impact of its use on patient and health service outcomes has been little studied. This recommendation aligns with the findings of this review, which reported no studies examining the outcomes of decision support and very few studies mentioning this component in any form.

Previous overviews on chronic conditions management [77, 78] suggested that further research should focus on assessing and reporting frequency and duration of nurse-led processes to determine an optimal time-related mode of delivery. In view of our results, we would argue that the primarily focus should be on integrating and reporting strategies related to decision support and clinical information system into the nursing processes. The CCM advocates for a concurrent implementation of all four components as their integration promotes resources and tools necessary for evidence-based care.

### Limitations

This umbrella review must be considered in light of certain limitations. First, the classification of reviews by activity was based on the main type of intervention reported by the authors. However, some intervention components overlapped. For example, case management regularly mentioned incorporating patient education, usually without a specific structure. Second, the use of an overview design provided a more complete picture of effectiveness from a large set of studies but has the limitation of excluding primary studies not included in a systematic review. Thus, some nursing processes may not have been included in this overview.

Third, forty-three primary studies overlapped in the systematic reviews. Apart from nurse prescribing, the effects of this overlap on outcomes were limited. A minimal number of these studies presented results on the same nursing process and outcome. Self-management support was assessed on diabetic populations in 5 of 7 reviews on this activity. Because this is the population primarily involved in the overlap of the primary studies, it is possible that the outcomes of this nursing activity are overrepresented. The current state of evidence does not suggest a clear guideline for addressing overlap. Because the number of multiple primary studies was minimal in all reviews and the purpose of our review was primarily descriptive, we decided not to exclude any review because of overlap. We suggest, however, that the findings of self-management support reported in this review should be interpreted in light of this overlap limitation.

There are also some limitations to be considered in relation to the choice of eligibility criteria for journals. On the recommendation of the specialist in bibliographic search methodology with whom we developed the search strategy, we chose to exclude reviews published before 2005. This choice was made to ensure that the findings were still relevant to nursing practice. Changes in best practice recommendations, in the role of the nurse over time, or in practice settings could influence the picture of nursing processes. However, this choice implies limitations in terms of the comprehensiveness of the research conducted and may have led to the exclusion of reviews that would have been relevant to our research questions. Finally, while we conducted a test for piloting the form used to extract the data, we did not assess inter-rater reliability among reviewers using a measure during the screening phase, which limits the reproducibility of this umbrella review.

## Conclusion

As care becomes more complex, there is a need to draw attention to the conditions that better support the implementation of nursing processes. Increased complexity requires advanced nursing competencies and a service delivery structure that supports effective coordination and provides management and communication tools [3, 4]. The integration of the CCM components would more effectively support flexible, consistent and personalized care to the evolving needs of patients with chronic conditions. This comprehensive overview of nursing processes aimed to provide a better understanding of how to support them in a broader way across the primary care continuum. The findings suggest that while the nature of clinical nursing processes is similar across settings and health conditions, their characteristics and their impact on outcomes are highly variable. As the need for an interdisciplinarity to primary care is widely emphasized, it is important that this approach not be considered solely from a clinical perspective. The organization of care and resources need to be designed to support the contributions of all professionals to optimize the full range of services provided to patients with chronic conditions.

### Electronic supplementary material

Below is the link to the electronic supplementary material.

Supplementary Material 1

Supplementary Material 2

Supplementary Material 3

Supplementary Material 4

## Data Availability

Data generated or analysed during this study are included in Additional Files. Datasets are available through the first author upon reasonable request.

## Abbreviations

AMSTAR: Assessing the Methodological Quality of Systematic Reviews
BA: Before-after
CADTH: Canadian Agency for Drugs and Technologies in Health
CCM: Chronic Care Model
CINAHL: Cumulative Index for Nursing and Allied Health Sciences
COPD: Chronic Obstructive Pulmonary Disease
CI: Confidence Interval
CVD: Cardiovascular disease
ED: Emergency department
GBP: British Pound Sterling
HF: Heart failure
HbA1c: Hemoglobin A1C
INESSS: Institut national d’excellence en santé et en services sociaux
MMAT: Mixed Methods Appraisal Tool
MD: Mean Difference
NRCT: Non-randomised controlled trial
NP Nurse: Practitioner
PRISMA: Preferred Reporting Items for Systematic Reviews and Meta-Analyses
RN: Registered Nurse
RCT: Randomized Controlled Trial
RR: Relative ratio
SBP: Systolic blood pressure
T2D: Type 2 Diabetes
USD: United States Dollar
