# Supplementary material for: Examining nursing processes in primary care settings using the Chronic Care Model: an umbrella review
Source: BMC Prim Care. 2023 Sep 4;24:176. doi: 10.1186/s12875-023-02089-3 (PMC10476383; doi:10.1186/s12875-023-02089-3)
Supplement: Supplementary file 2 — Supplementary Material 2 [file 12875_2023_2089_MOESM2_ESM.docx]

**Additional File 2**

Title: Sample of the research strategy

Description: An example of the detailed search strategy used for the MEDLINE database.

| **MEDLINE (Ovid)** | |
| --- | --- |
|  | |
| **Limits : 2005- ; English and French** | |
| 1 | *Chronic Disease/ OR *Multiple Chronic Conditions/ OR Multimorbidity/ OR Comorbidity/ |
| 2 | (multidisease? OR multi-disease? OR (multiple ADJ (ill$ OR disease? OR condition? OR syndrom$ OR disorder?))).ti,ab. |
| 3 | (chronic$ ADJ3 (disease? OR ill$ OR care OR condition? OR disorder$ OR health$ OR medication$ OR syndrom$ OR symptom$)).ti,ab. |
| 4 | (comorbid$ OR co-morbid$ OR multimorbid$ OR multi-morbid$).ti,ab. |
| 5 | OR/1-4 |
| 6 | exp Arthritis Rheumatoid/ OR exp Asthma/ OR exp Cerebrovascular Disorders/ OR exp Diabetes Mellitus/ OR exp Epilepsy/ OR exp Heart Diseases/ OR exp HIV Infections/ OR exp Hyperlipidemia/ OR exp Hypertension/ OR exp Kidney Diseases/ OR exp Liver Diseases/ OR exp Mental Disorders/ OR exp Neoplasms/ OR exp Osteoporosis/ OR exp Pulmonary Disease Chronic Obstructive/ OR exp Thyroid Diseases/ |
| 7 | (asthma$ OR cancer? OR diabet$ OR epileps$ OR hypertens$ OR high blood pressure? OR neoplasm? OR osteoporosis OR rheumatoid arthritis OR seizure?).ti,ab. |
| 8 | (((heart OR cardiac OR cardiovascular OR coronary) ADJ (disease? OR disorder? OR failure)) OR arrythmia?).ti,ab. |
| 9 | ((cerebrovascular OR vascular OR carotoid$ OR arter$) ADJ (disorder? OR disease?)).ti,ab. |
| 10 | (copd OR (pulmonary ADJ2 (disease? OR disorder?))).ti,ab. |
| 11 | (hyperlipidem$ OR hypercholesterolemia$ OR hypertriglyceridemia$).ti,ab. |
| 12 | ((thyroid ADJ (disease? OR disorder)) OR hyperthyroid$ OR hypothyroid$).ti,ab. |
| 13 | (((mental OR anxiety OR mood OR psychological OR sleep) ADJ (disease? OR disorder?)) OR ((substance OR drug OR marijuana OR cocaine OR Amphetamine) ADJ2 abuse) OR depression OR schizophren$ OR psychos$ OR addiction?).ti,ab. |
| 14 | (HIV OR acquired immune$ deficiency syndrome? OR (aids ADJ (associated OR related OR arteritis))).ti,ab. |
| 15 | ((kidney OR liver) ADJ (disease? OR disorder?)).ti,ab. |
| 16 | OR/6-15 |
| 17 | ((coocur$ OR co-ocur$ OR coexist$ OR co-exist$ OR multipl$) ADJ3 (disease? OR ill$ OR care OR condition? OR disorder$ OR health$ OR medication$ OR symptom$ OR syndrom$)).ti,ab. |
| 18 | chronic$.ti,ab,hw. |
| 19 | OR/17-18 |
| 20 | 16 AND 19 |
| 21 | 5 OR 20 |
| 22 | Comprehensive Health Care/ |
| 23 | (comprehensive health care OR comprehensive healthcare).ti,ab. |
| 24 | OR/22-23 |
| 25 | 21 AND 24 |
| 26 | Patient-Centered Care/ OR Polypharmacy/ |
| 27 | (multi-drug therapy OR multidrug therapy OR multiple drug therapy OR multiple drug treatment OR multiple pharmacotherapy OR polymedication OR polypharmac$ OR polypragmas$).ti,ab. |
| 28 | (medical home OR patient-centered care OR patient-centered nursing OR patient focused care).ti,ab. |
| 29 | OR/26-28 |
| 30 | Delivery of Health Care/ |
| 31 | (delivery of health care OR delivery of healthcare OR health care delivery OR health care supply OR health care systems OR healthcare delivery OR healthcare supply OR healthcare systems).ti,ab. |
| 32 | OR/30-31 |
| 33 | 21 AND 29 AND 32 |
| 34 | *Primary Care Nursing/ |
| 35 | primary care nurs*.ti,ab. |
| 36 | OR/34-35 |
| 37 | 21 AND 36 |
| 38 | Professional Practice/ |
| 39 | 21 AND 38 |
| 40 | Primary Health Care/ |
| 4 | (first line care OR primary ADJ2 (care? OR medical$ OR health$ OR clinic$ OR practitioner? OR doctor?)).ti,ab. |
| 42 | OR/40-41 |
| 43 | 21 AND 42 |
| 44 | 29 AND 43 |
| 45 | 32 AND 43 |
| 46 | Education, Nursing/ |
| 47 | nursing education.ti,ab. |
| 48 | OR/46-47 |
| 49 | *Advance Directives/ OR exp *Education, Continuing/ OR *Feedback/ OR *Guideline Adherence/ OR *Pamphlets/ OR *Reminder Systems/ |
| 50 | (education$ ADJ2 (intervention? OR meeting? OR program$ OR session? OR strateg$ OR visit? OR workshop?)).tw. |
| 51 | (behavio?r$ ADJ2 intervention?).tw. |
| 52 | (academic detailing OR algorithm? OR booklet? OR chart review$ OR compliance OR consensus conference? OR facilitator? OR feedback OR leaflet? OR marketing OR outreach OR poster OR posters OR practice guideline? OR prompter? OR prompting OR reminder?).tw. |
| 53 | ((oral OR printed OR written) ADJ information).tw. |
| 54 | (information$ ADJ2 campaign).tw. |
| 55 | (education$ ADJ1 (material? OR method?)).tw. |
| 56 | ((education$ OR influential OR opinion) ADJ1 leader?).tw. |
| 57 | (guideline? ADJ2 (disseminat$ OR distribut$ OR effect? OR impact OR introduc$ OR issu$)).tw. |
| 58 | ((compar$ OR effect? OR evaluat$ OR impact OR introduc$) ADJ2 training program$).tw. |
| 59 | (recall ADJ2 system$).tw. |
| 60 | ((chart? OR effect? OR impact OR records) ADJ2 audit).tw. |
| 61 | OR/49-60 |
| 62 | 48 OR 61 |
| 63 | 43 AND 62 |
| 64 | (best practice* OR gold standard).ti,ab. |
| 65 | 43 AND 64 |
| 66 | *Nurse Clinicians/ OR *Nurse Midwives/ OR *Nurse Practitioners/ OR Nurses/ |
| 67 | (nurse ADJ (clinician? OR midwi$ OR practitioner? OR rehabilitator?)).tw. |
| 68 | (convalescence home* OR convalescence hospital OR extended care facility OR nursing home*).tw. |
| 69 | *Ambulatory Care/ OR exp *Ambulatory Care Facilities/ OR exp *Patient Care Planning/ OR *Patient Care Team/ OR *Pharmacists/ |
| 70 | clinical pharmacist?.tw. |
| 71 | (team? ADJ2 (assessment OR care OR consultation OR treatment)).tw. |
| 72 | (integrat$ ADJ2 (care OR service?)).tw. |
| 73 | (care ADJ2 (continuity OR coordinat$ OR program$)).tw. |
| 74 | (case ADJ1 management).tw. |
| 75 | OR/66-74 |
| 76 | *Aftercare/ OR *Community Health Nursing/ OR *Day Care/ OR *Home Care Services/ OR *Hospices/ OR *House Calls/ OR Nursing Homes/ OR *Office Visits/ |
| 77 | ((chang$ ADJ1 location?) OR day surgery OR domiciliary OR (home ADJ1 treat$)).tw. |
| 78 | *Medical Records/ OR *Medical Records Systems, Computerized/ |
| 79 | (information ADJ2 (management OR system?)).tw. |
| 80 | exp *Health Services Misuse/ OR *Peer Review/ OR *Utilization Review/ |
| 81 | OR/76-80 |
| 82 | *Length of Stay/ OR *"Physician’s Practice Patterns"/ OR *"Process Assessment (Health Care)"/ OR *Program Evaluation/ |
| 83 | (discharge planning OR (early ADJ1 discharg$) OR offset OR quality assurance OR triage).tw. |
| 84 | exp *"Referral and Consultation"/ |
| 85 | *Drug Therapy, Computer Assisted/ OR *Health Maintenance Organizations/ OR *Medical History Taking/ OR *Telephone/ |
| 86 | ((hospital? ADJ1 merg$) OR managed care OR near patient testing OR (physician patient ADJ (interaction? OR relationship?))).tw. |
| 87 | OR/82-86 |
| 88 | 75 OR 81 OR 87 |
| 89 | 43 AND 88 |
| 90 | 25 OR 33 OR 37 OR 39 |
| 91 | 44 OR 45 OR 63 OR 65 OR 89 |
| 92 | 90 OR 91 |
