# Supplementary material for: Examining nursing processes in primary care settings using the Chronic Care Model: an umbrella review
Source: BMC Prim Care. 2023 Sep 4;24:176. doi: 10.1186/s12875-023-02089-3 (PMC10476383; doi:10.1186/s12875-023-02089-3)
Supplement: Supplementary file 3 — Supplementary Material 3 [file 12875_2023_2089_MOESM3_ESM.docx]

**Additional File 3**

Title: List of excluded full-texts and reasons of exclusion

Description: List of the 112 excluded articles and their reason for exclusion.

| Article references | Reason for exclusion |
| --- | --- |
| 1. Allen, J., Hutchinson, A. M., Brown, R. et Livingston, P. M. (2014). Quality care outcomes following transitional care interventions for older people from hospital to home: a systematic review. *BMC Health Services Research, 14*, 346. | No analysis specific to primary care |
| 1. Anand, T. N., Joseph, L. M., Geetha, A. V., Prabhakaran, D. et Jeemon, P. (2019). Task sharing with non-physician health-care workers for management of blood pressure in low-income and middle-income countries: a systematic review and meta-analysis. The Lancet Global Health, 7(6), e761-e771. | No analysis specific to primary care |
| 1. Anderson, L., Brown, P. R. J., Clark, A. M., Dalal, H., Rossau, K. H., Bridges, C. et Taylor, R. S. (2018). Patient education in the management of coronary heart disease. Cochrane Database of Systematic Reviews(10). | No analysis specific to primary care |
| 1. Health Quality, O. (2013). Specialized nursing practice for chronic disease management in the primary care setting: an evidence-based analysis. Ontario Health Technology Assessment Series, 13(10), 1-66. | Not a systematic review |
| 1. Magwood, G. S., Nichols, M., Jenkins, C., Logan, A., Qanungo, S., Zigbuo-Wenzler, E. et Ellis, C. J. (2020). Community-Based Interventions for Stroke Provided by Nurses and Community Health Workers: A Review of the Literature. The Journal of neuroscience nursing : journal of the American Association of Neuroscience Nurses, 52(4), E7-E8. | Not a systematic review |
| 1. Anthony, B. F., Surgey, A., Hiscock, J., Williams, N. H. et Charles, J. M. (2019). General medical services by non-medical health professionals: a systematic quantitative review of economic evaluations in primary care. British Journal of General Practice, 69(682), e304-e313. | No analysis specific to a nursing activity |
| 1. Aubin, M., Giguere, A., Martin, M., Verreault, R., Fitch, I. M., Kazanjian, A. et Carmichael, P. (2012). Interventions to improve continuity of care in the follow-up of patients with cancer. Cochrane Database of Systematic Reviews(7). | No analysis specific to a nursing activity |
| 1. Aydede, S. K., Komenda, P., Djurdjev, O. et Levin, A. (2014). Chronic kidney disease and support provided by home care services: a systematic review. BMC Nephrology, 15, 118. | No analysis specific to primary care |
| 1. Baggott, R., Scott, D., Sturt, J., Bosworth, A., Parker, L., Georgopoulou, S. et Lempp, H. (2019). What is the value, impact and role of nurses in rheumatology outpatient care? Rheumatology (United Kingdom), 58 (Supplement 3), iii102-iii103. | Not a systematic review |
| 1. Bailey, D. E., Jr. et Zucker, D. M. (2020). Supportive Interventions During Treatment of Chronic Hepatitis C: A Review of the Literature. Gastroenterology Nursing, 43(5), E172-E183. | No analysis specific to a nursing activity |
| 1. Barrett, K. et Chang, Y. P. (2016). Behavioral Interventions Targeting Chronic Pain, Depression, and Substance Use Disorder in Primary Care. Journal of Nursing Scholarship, 48(4), 345-353. doi: 10.1111/jnu.12213 | No analysis specific to a nursing activity |
| 1. Belling, R., McLaren, S. et Woods, L. (2010). Specialist nursing interventions for inflammatory bowel disease. Cochrane Database of Systematic Reviews(1). | No analysis specific to primary care |
| 1. Benjenk, I. et Chen, J. (2018). Effective mental health interventions to reduce hospital readmission rates: a systematic review. Journal of Hospital Management and Health Policy, 2. | Not a systematic review |
| 1. Berger, Z., Hsiao, Y. L., Riese, V. G., Walker, R. K., Davidson, P., Pollack, C. E. et Snyder, C. F. (2016). Primary care and outcomes in adult patients with cancer: A systematic review. Journal of General Internal Medicine, 1), S360. | Not a systematic review |
| 1. Bhanbhro, S., Drennan, V. M., Grant, R. et Harris, R. (2011). Assessing the contribution of prescribing in primary care by nurses and professionals allied to medicine: a systematic review of literature. BMC Health Services Research, 11, 330. | Not chronic condition specific |
| 1. Bradshaw, T., Lovell, K. et Harris, N. (2005). Healthy living interventions and schizophrenia: a systematic review. Journal of Advanced Nursing, 49(6), 634-654. | No analysis specific to primary care |
| 1. Browall, M., Forsberg, C. et Wengstrom, Y. (2017). Assessing patient outcomes and cost-effectiveness of nurse-led follow-up for women with breast cancer - have relevant and sensitive evaluation measures been used? Journal of Clinical Nursing, 26(13-14), 1770-1786. | No analysis specific to primary care |
| 1. Bunn, F., Goodman, C., Pinkney, E. et Drennan, V. M. (2016). Specialist nursing and community support for the carers of people with dementia living at home: an evidence synthesis. Health & Social Care in the Community, 24(1), 48-67. | Not a systematic review with primary studies |
| 1. Butterworth, J. E., Hays, R., McDonagh, S. T., Richards, S. H., Bower, P. et Campbell, J. (2019). Interventions for involving older patients with multi-morbidity in decision-making during primary care consultations. Cochrane Database of Systematic Reviews, 10(10), 28. | No analysis specific to a nursing activity |
| 1. Case, R., Haynes, D., Holaday, B. et Parker, V. G. (2010). Evidence-based nursing: the role of the advanced practice registered nurse in the management of heart failure patients in the outpatient setting. DCCN - Dimensions of Critical Care Nursing, 29(2), 57-62; quiz 63-54. | Not a systematic review |
| 1. Centre for Evidence-Based Nursing South, A. (2006). Nurse-led cardiac clinics for adults with coronary heart disease. Australian Nursing Journal, 14(6), 25-28. | Not a systematic review |
| 1. Horrocks, S., Anderson, E. et Salisbury, C. (2002). Systematic review of whether nurse practitioners working in primary care can provide equivalent care to doctors (Structured abstract). Database of Abstracts of Reviews of Effects(2). | Published before 2005 |
| 1. Sargent, G. M., Forrest, L. E. et Parker, R. M. (2012). Nurse delivered lifestyle interventions in primary health care to treat chronic disease risk factors associated with obesity: a systematic review. Obesity Reviews, 13(12), 1148-1171. doi: https://doi.org/10.1111/j.1467-789X.2012.01029.x | Not chronic condition specific |
| 1. Heideman, J., van Rijswijk, E., van Lin, N., de Loos, S., Laurant, M., Wensing, M., . . . Grol, R. (2005). Interventions to improve management of anxiety disorders in general practice: a systematic review. Br J Gen Pract, 55(520), 867-874. | No analysis specific to a nursing activity |
| 1. Stuck, A. E., Egger, M., Hammer, A., Minder, C. E. et Beck, J. C. (2002). Home visits to prevent nursing home admission and functional decline in elderly people: systematic review and meta-regression analysis. JAMA, 287(8), 1022-1028. doi: 10.1001/jama.287.8.1022 | Published before 2005 |
| 1. Worrall, G. et Knight, J. (2006). Continuity of care for older patients in family practice: how important is it? Canadian family physician Medecin de famille canadien, 52(6), 754-755. | No analysis specific to a nursing activity |
| 1. Noordman, J., van der Weijden, T. et van Dulmen, S. (2012). Communication-related behavior change techniques used in face-to-face lifestyle interventions in primary care: a systematic review of the literature. Patient Educ Couns, 89(2), 227-244. doi: 10.1016/j.pec.2012.07.006 | Not chronic condition specific |
| 1. Chandra Sekran, A. M., Ajay, V. S., Kinra, S. et Prabhakaran, D. (2014). Nurse care interventions (NCI) for the management of type 2 diabetes mellitus in primary care settings-a systematic review and meta-analysis of randomized, controlled trials. Diabetes, 1), A309. | Not a systematic review |
| 1. Christensen, H., Griffiths, K. M., Gulliver, A., Clack, D., Kljakovic, M. et Wells, L. (2008). Models in the delivery of depression care: a systematic review of randomised and controlled intervention trials. BMC Family Practice, 9, 25. | No analysis specific to a nursing activity |
| 1. Coffey, A., Mulcahy, H., Savage, E., Fitzgerald, S., Bradley, C., Benefield, L. et Leahy-Warren, P. (2017). Transitional care interventions: Relevance for nursing in the community. Public health nursing (Boston, Mass.), 34(5), 454-460. | Not chronic condition specific |
| 1. Coolbrandt, A., Van Der Elst, E., Wildiers, H., Aertgeerts, B., Dierckx de Casterle, B., Van Achterberg, T., . . . Milisen, K. (2013). Complex nursing interventions aimed at reducing chemotherapyrelated symptom burden: A systematic review of their characteristics and effectiveness. Supportive Care in Cancer, 1), S160-S161. | No analysis specific to primary care |
| 1. Cruz-Cobo, C. et Santi-Cano, M. J. (2020). Efficacy of Diabetes Education in Adults With Diabetes Mellitus Type 2 in Primary Care: A Systematic Review. Journal of nursing scholarship : an official publication of Sigma Theta Tau International Honor Society of Nursing, 52(2), 155-163. | No analysis specific to a nursing activity |
| 1. Cutler, S. (2018). Effectiveness of Group Self-Management Interventions for Persons with Chronic Conditions: A Systematic Review. MEDSURG Nursing, 27(6), 359-367. | No analysis specific to primary care |
| 1. De Thurah, A., Appel Esbensen, B., Roelsgaard, I. K., Frandsen, T. F. et Primdahl, J. (2016). The effect of nurse-led follow-up in rheumatoid arthritis. A systematic review and meta-analysis of randomized controlled trails. Arthritis and Rheumatology, 68 (Supplement 10), 1484-1485. | No analysis specific to primary care |
| 1. Dennis, S., May, J., Perkins, D., Zwar, N., Sibbald, B. et Hasan, I. (2009). What evidence is there to support skill mix changes between GPs, pharmacists and practice nurses in the care of elderly people living in the community? Australia & New Zealand Health Policy, 6, 23. | No analysis specific to a nursing activity |
| 1. Fahey, T., Schroeder, K. et Ebrahim, S. (2005). Educational and organisational interventions used to improve the management of hypertension in primary care: A systematic review. British Journal of General Practice, 55(520), 875-882. | No analysis specific to a nursing activity |
| 1. Glynn, L. G., Murphy, A. W., Smith, S. M., Schroeder, K. et Fahey, T. (2010). Interventions used to improve control of blood pressure in patients with hypertension. Cochrane Database Syst Rev(3), CD005182. doi: 10.1002/14651858.CD005182.pub4 | No analysis specific to a nursing activity |
| 1. Faruqi, N., Joshi, C., Dennis, S., Lloyd, J., Taggart, J., Spooner, C. et Harris, M. (2013). What health literacy interventions are effective in the primary healthcare settings in weight loss management - A systematic review. Obesity Research and Clinical Practice, 2), e99. | No analysis specific to a nursing activity |
| 1. Fergenbaum, J., Bermingham, S., Krahn, M., Alter, D. et Demers, C. (2015). Care in the Home for the Management of Chronic Heart Failure. Journal of Cardiovascular Nursing, S44-51. doi: 10.1097/JCN.0000000000000235 | No analysis specific to primary care |
| 1. Ferguson, S., Swan, M. et Smaldone, A. (2015). Does diabetes self-management education in conjunction with primary care improve glycemic control in Hispanic patients? A systematic review and meta-analysis. The Diabetes educator, 41(4), 472-484. | No analysis specific to a nursing activity |
| 1. Fleming, P. et Godwin, M. (2008). Lifestyle interventions in primary care: systematic review of randomized controlled trials. Canadian Family Physician, 54(12), 1706-1713. | Not chronic condition specific |
| 1. Gallagher, H., de Lusignan, S., Harris, K. et Cates, C. (2010). Quality-improvement strategies for the management of hypertension in chronic kidney disease in primary care: a systematic review. British Journal of General Practice, 60(575), e258-265. | No analysis specific to a nursing activity |
| 1. Gibson, C., Goeman, D. et Pond, D. (2020). What is the role of the practice nurse in the care of people living with dementia, or cognitive impairment, and their support person(s)?: a systematic review. BMC Family Practice, 21(1), 141. | No patient-related or care delivery outcomes |
| 1. Gitlin, L. (2012). Interventions to promote well-being for people with dementia and their family caregivers. Alzheimer's and Dementia, 1), P229-P230. | Not a systematic review |
| 1. Graven, C., Brock, K., Hill, K. et Joubert, L. (2011). Are rehabilitation and/or care co-ordination interventions delivered in the community effective in reducing depression, facilitating participation and improving quality of life after stroke? Disability and rehabilitation, 33(17-18), 1501-1520. | No analysis specific to a nursing activity |
| 1. Griffiths, P., Richardson, A. et Blackwell, R. (2012). Outcomes sensitive to nursing service quality in ambulatory cancer chemotherapy: Systematic scoping review. European Journal of Oncology Nursing, 16(3), 238-246. | No analysis specific to primary care |
| 1. Gurusamy, J., Gandhi, S., Damodharan, D., Ganesan, V. et Palaniappan, M. (2018). Exercise, diet and educational interventions for metabolic syndrome in persons with schizophrenia: A systematic review. Asian Journal of Psychiatry, 36, 73-85. | No analysis specific to primary care |
| 1. Halcomb, E., Moujalli, S., Griffiths, R. et Davidson, P. (2007). Effectiveness of general practice nurse interventions in cardiac risk factor reduction among adults. JBI Library of Systematic Reviewis, 5(7), 407-453. | Not chronic condition specific |
| 1. Hanlon, P., Yeoman, L., Gibson, L., Esiovwa, R., Williamson, A. E., Mair, F. S. et Lowrie, R. (2018). A systematic review of interventions by healthcare professionals to improve management of non-communicable diseases and communicable diseases requiring long-term care in adults who are homeless. BMJ Open, 8 (4) (no pagination)(e020161). | No analysis specific to a nursing activity |
| 1. Harrison, S. L., Janaudis-Ferreira, T., Brooks, D., Desveaux, L. et Goldstein, R. S. (2015). Self-management following an acute exacerbation of COPD: a systematic review. Chest, 147(3), 646-661. | No analysis specific to a nursing activity |
| 1. Heise, B. A. et van Servellen, G. (2014). The nurse's role in primary care antidepressant medication adherence. Journal of Psychosocial Nursing & Mental Health Services, 52(4), 48-57. | Not a systematic review |
| 1. Hoeg, B. L., Bidstrup, P. E., Karlsen, R. V., Friberg, S. A., Albieri, V., Dalton, S. O., . . . Johansen, C. (2019). Follow-up strategies following completion of primary cancer treatment in adult cancer survivors. Cochrane Database of Systematic Reviews(11). | No analysis specific to primary care |
| 1. Howcroft, M., Walters, H. E., WoodBaker, R. et Walters, A. E. J. (2016). Action plans with brief patient education for exacerbations in chronic obstructive pulmonary disease. Cochrane Database of Systematic Reviews(12). | No analysis specific to a nursing activity |
| 1. Hudon, C., Fortin, M. et Soubhi, H. (2008). Single risk factor interventions to promote physical activity among patients with chronic diseases: systematic review. Canadian Family Physician, 54(8), 1130-1137. | No analysis specific to a nursing activity |
| 1. Igai, Y. (2019). Effectiveness of non-pharmacological nursing interventions to improve the quality of life of patients with idiopathic pulmonary fibrosis: A systematic review. Japan journal of nursing science : JJNS, 16(3), 241-252. | No analysis specific to primary care |
| 1. The Joanna Briggs Institute Best Practice Information Sheet: Nurse-led interventions to reduce cardiac risk factors in adults. (2010). Nurs Health Sci, 12(3), 288-291. doi: 10.1111/j.1442-2018.2010.00548.x | Not a systematic review |
| 1. Johnson, J. M. et Carragher, R. (2018). Interprofessional collaboration and the care and management of type 2 diabetic patients in the Middle East: A systematic review. Journal of Interprofessional Care, 32(5), 621-628. | No analysis specific to a nursing activity |
| 1. Joo, J. Y. et Liu, M. F. (2019). Case management effectiveness for managing chronic illnesses in Korea: a systematic review. International Nursing Review, 66(1), 30-42. | Context-specific effects |
| 1. Joo, J. Y. et Huber, D. L. (2019). Case Management Effectiveness on Health Care Utilization Outcomes: A Systematic Review of Reviews. Western Journal of Nursing Research, 41(1), 111-133. doi: 10.1177/0193945918762135 | Not a systematic review of primary studies |
| 1. Kai Lun Gan, J., Brammer, J. D. et Creedy, D. K. (2011). Effectiveness of educational interventions to promote oral hypoglycaemic adherence in adults with Type 2 diabetes: a systematic review. JBI Library of Systematic Reviews, 9(9), 269-312. doi: 10.11124/jbisrir-2011-92 | No analysis specific to a nursing activity |
| 1. Keleher, H., Parker, R., Abdulwadud, O. et Francis, K. (2009). Systematic review of the effectiveness of primary care nursing. International Journal of Nursing Practice, 15(1), 16-24. | No description of nursing activity |
| 1. Khalil, H., Bell, B., Chambers, H., Sheikh, A. et Avery, A. J. (2017). Professional, structural and organisational interventions in primary care for reducing medication errors. Cochrane Database of Systematic Reviews(10). | No analysis specific to a nursing activity |
| 1. Khan, A. H., Tolley, C. L., Bimpong, K. A. et Slight, S. P. (2019). A systematic review identifying the impact of patient decision aids in older patients with problematic polypharmacy. International Journal of Pharmacy Practice, 27 (Supplement 2), 18-19. | Not a published systematic review |
| 1. Kim, K., Choi, J. S., Choi, E., Nieman, C. L., Joo, J. H., Lin, F. R., . . . Han, H. R. (2016). Effects of Community-Based Health Worker Interventions to Improve Chronic Disease Management and Care Among Vulnerable Populations: A Systematic Review. American journal of public health, 106(4), e3-e28. | No analysis specific to a nursing activity |
| 1. Kruis, A. L., Smidt, N., Assendelft, J. J. W., Gussekloo, J., Boland, R. S. M., Ruttenvan Molken, M. et Chavannes, N. H. (2013). Integrated disease management interventions for patients with chronic obstructive pulmonary disease. Cochrane Database of Systematic Reviews(10). | No analysis specific to a nursing activity |
| 1. Kyriakou, M., Kaloyirou, F., Mantle, R. et Deaton, C. (2018). Do heart failure management programmes improve outcomes for patients with heart failure with preserved ejection fraction? European Journal of Cardiovascular Nursing, 17 (1 Supplement 1), 20-21. | No analysis specific to a nursing activity |
| 1. Laurant, M., van der Biezen, M., Wijers, N., Watananirun, K., Kontopantelis, E. et van Vught, A. J. (2018). Nurses as substitutes for doctors in primary care. Cochrane Database Syst Rev, 7(7), CD001271. doi: 10.1002/14651858.CD001271.pub3 | No analysis specific to a nursing activity |
| 1. Lawton, K., Royals, K., Carson-Chahhoud, K. V., Campbell, F. et Smith, B. J. (2018). Nurse-led versus doctor-led care for bronchiectasis. Cochrane Database of Systematic Reviews(6). | No analysis specific to primary care |
| 1. Lee, D. C. A., Tirlea, L. et Haines, T. P. (2020). Non-pharmacological interventions to prevent hospital or nursing home admissions among community-dwelling older people with dementia: A systematic review and meta-analysis. Health & Social Care in the Community, 28(5), 1408-1429. | No analysis specific to a nursing activity |
| 1. Leonard, B. (2006). Review: existing evidence does not support nurse led interventions in chronic obstructive pulmonary disease. Evidence Based Nursing, 9(2), 56-56. | Not a systematic review |
| 1. Lewis, R., Neal, R. D., Williams, N. H., France, B., Wilkinson, C., Hendry, M., . . . Weller, D. (2009). Nurse-led vs. conventional physician-led follow-up for patients with cancer. Journal of Advanced Nursing, 65(4), 706-723. | No analysis specific to primary care |
| 1. Lupari, M., Coates, V., Adamson, G. et Crealey, G. E. (2011). 'We're just not getting it right'- how should we provide care to the older person with multi-morbid chronic conditions? Journal of Clinical Nursing (John Wiley & Sons, Inc.), 20(9-10), 1225-1235. doi: 10.1111/j.1365-2702.2010.03620.x | Not a systematic review |
| 1. Martinez-Gonzalez, N. A., Djalali, S., Tandjung, R., Huber-Geismann, F., Markun, S., Wensing, M. et Rosemann, T. (2014). Substitution of physicians by nurses in primary care: a systematic review and meta-analysis. BMC Health Services Research, 14, 214. | Not chronic condition specific |
| 1. Matthys, E., Remmen, R. et Van Bogaert, P. (2017). An overview of systematic reviews on the collaboration between physicians and nurses and the impact on patient outcomes: what can we learn in primary care? BMC Family Practice, 18(1), 110. | No analysis specific to a nursing activity |
| 1. Mills, K. T., Obst, K. M., Shen, W., Molina, S., Zhang, H. J., He, H. et He, J. (2018). Role of providers in implementation of blood pressure control strategies in patients with hypertension. Circulation. Conference: American Heart Association's Epidemiology and Prevention/Lifestyle and Cardiometabolic Health, 137(Supplement 1). | No analysis specific to a nursing activity |
| 1. Mulligan, H., Wilkinson, A., Chen, D., Nijhof, C., Kwan, N., Lindup, A. et Dalton, S. (2019). Components of community rehabilitation programme for adults with chronic conditions: A systematic review. International Journal of Nursing Studies, 97, 114-129. | No analysis specific to a nursing activity |
| 1. Munn, Z. (2010). Review summaries: Evidence for nursing practice. Promoting and supporting self-care management for adults living in the community with physical chronic illness: a systematic review of the effectiveness and meaningfulness of the patient-practitioner encounter. Journal of Advanced Nursing (John Wiley & Sons, Inc.), 66(1), 17-18. doi: 10.1111/j.1365-2648.2009.05187.x | Not a published systematic review |
| 1. Norful, A., Martsolf, G., de Jacq, K. et Poghosyan, L. (2017). Utilization of registered nurses in primary care teams: A systematic review. International Journal of Nursing Studies, 74, 15-23. | Not chronic condition specific |
| 1. Nurmatov, U., Buckingham, S., Kendall, M., Murray, S. A., White, P., Sheikh, A. et Pinnock, H. (2012). Effectiveness of holistic interventions for people with severe chronic obstructive pulmonary disease: systematic review of controlled clinical trials. PLoS ONE [Electronic Resource], 7(10), e46433. | No analysis specific to primary care |
| 1. O’Brien, N., Hong, Q. N., Law, S., Massoud, S., Carter, A., Kaida, A., . . . de Pokomandy, A. (2018). Health System Features That Enhance Access to Comprehensive Primary Care for Women Living with HIV in High-Income Settings: A Systematic Mixed Studies Review. AIDS Patient Care & Stds, 32(4), 129-148. doi: 10.1089/apc.2017.0305 | No analysis specific to a nursing activity |
| 1. O'Neill, I., Gale, C. P., McCallum, A., McIntyre, H., Squire, I. et Cherif, M. (2017). Impact of mode of delivery of disease management programmes on clinical outcomes among patients following hospitalised heart failure: A systematic review and meta-analysis. European Journal of Heart Failure, 19 (Supplement 1), 227. | Not a published systematic review |
| 1. Ogedegbe, G., Gyamfi, J., Plange-Rhule, J., Surkis, A., Rosenthal, D. M., Airhihenbuwa, C., . . . Cooper, R. (2014). Task shifting interventions for cardiovascular risk reduction in low-income and middle-income countries: a systematic review of randomised controlled trials. BMJ Open, 4(10), e005983. | No analysis specific to a nursing activity |
| 1. Poitras, M.-E., Chouinard, M.-C., Fortin, M., Girard, A. et Gallagher, F. (2016). Les activités des infirmières œuvrant en soins de première liane auprès des personnes atteintes de maladies chroniques : une revue systématique de la littérature. Recherche en Soins Infirmiers, 126(3), 24-37. doi: 10.3917/rsi.126.0024 | No patient-related or care delivery outcomes |
| 1. Prasad, S. S., Potter, M., Keely, S., Talley, N. J., Walker, M. M. et Kairuz, T. (2020). Roles of healthcare professionals in the management of chronic gastrointestinal diseases with a focus on primary care: A systematic review. Jgh Open, 4(2), 221-229. | No patient-related or care delivery outcomes |
| 1. Prencipe, G. P., Mele, A., Migliara, G., Nardi, A., Massimi, A., Rega, M. L., . . . Damiani, G. (2019). Impact of educational interventions on behavioral changes in chronic patients educated by nurses in community setting. Evidence from a systematic review. Journal of Preventive Medicine and Hygiene, 60 (3 Supplement 1), E372. | Not a published systematic review |
| 1. Proia, K. K., Thota, A. B., Njie, G. J., Finnie, R. K. C., Hopkins, D. P., Mukhtar, Q., . . . Cooksey, T. (2014). Team-based care and improved blood pressure control: A community guide systematic review. American Journal of Preventive Medicine, 47(1), 86-99. | No analysis specific to a nursing activity |
| 1. Rees, S. et Williams, A. (2009). Promoting and supporting self-management for adults living in the community with physical chronic illness: A systematic review of the effectiveness and meaningfulness of the patient-practitioner encounter. JBI Library of Systematic Reviewis, 7(13), 492-582. | No analysis specific to a nursing activity |
| 1. Reilly, R., Evans, K., Gomersall, J., Gorham, G., Peters, M. D., Warren, S., . . . Brown, A. (2016). Effectiveness, cost effectiveness, acceptability and implementation barriers/enablers of chronic kidney disease management programs for Indigenous people in Australia, New Zealand and Canada: a systematic review of mixed evidence. BMC Health Services Research, 16, 119. | No analysis specific to a nursing activity |
| 1. Renders, C. M., Valk, G. D., Griffin, S., Wagner, E. H., Eijk, J. T. et Assendelft, W. J. (2001). Interventions to improve the management of diabetes mellitus in primary care, outpatient and community settings. Cochrane Database Syst Rev, 2000(1), CD001481. doi: 10.1002/14651858.cd001481 | Published before 2005 |
| 1. Santomassino, M., Costantini, G. D., McDermott, M., Primiano, D., Slyer, J. T. et Singleton, J. K. (2012). A systematic review on the effectiveness of continuity of care and its role in patient satisfaction and decreased hospital readmissions in the adult patient receiving home care services. JBI Library of Systematic Reviewis, 10(21), 1214-1259. | Not chronic condition specific |
| 1. Saxena, S., Misra, T., Car, J., Netuveli, G., Smith, R. et Majeed, A. (2007). Systematic review of primary healthcare interventions to improve diabetes outcomes in minority ethnic groups. Journal of Ambulatory Care Management, 30(3), 218-230. | No analysis specific to a nursing activity |
| 1. Schnitker, L., Novic, A., Arendts, G., Carpenter, C. R., LoGiudice, D., Caplan, G. A., . . . Beattie, E. (2020). Prevention of Delirium in Older Adults With Dementia: A Systematic Literature Review. Journal of Gerontological Nursing, 46(10), 43-54. | No analysis specific to primary care |
| 1. Shah, H. A. et Abu-Amara, M. (2013). Education provides significant benefits to patients with hepatitis B virus or hepatitis C virus infection: a systematic review. Clinical Gastroenterology & Hepatology, 11(8), 922-933. | No analysis specific to a nursing activity |
| 1. Shin, G. I., Woo, Y. S., Park, D. S. et Park, H. Y. (2019). The Randomized Controlled Trials (Rcts) with Community-Based Intervention for Patients with Dementia: A Systematic Review. Alzheimer's and Dementia, 15 (7 Supplement), P1594-P1595. | Not a published systematic review |
| 1. Siying, L., Kim, E. A. N. et Moon Fai, C. (2010). The effectiveness of nurse-led telephone consultations on reducing emotional distress in community-dwelling adults with cancer: A systematic review. JBI Database Of Systematic Reviews And Implementation Reports, 8(34 Supplement), S519-S550. | Not a published systematic review |
| 1. Slyer, J. T. et Ferrara, L. R. (2013). The effectiveness of group visits for patients with heart failure on knowledge, quality of life, self-care, and readmissions: A systematic review. JBI Database Of Systematic Reviews And Implementation Reports, 11(7), 58-81. | No analysis specific to a nursing activity |
| 1. Smith, S. M., Wallace, E., O'Dowd, T. et Fortin, M. (2017). Interventions for improving outcomes in patients with multimorbidity in primary care and community settings. Cochrane Database of Systematic Reviews(9). | No analysis specific to a nursing activity |
| 1. Song, Y., Lu, H., Chen, H., Geng, G. et Wang, J. (2014). Mindfulness intervention in the management of chronic pain and psychological comorbidity: A meta-analysis. International Journal of Nursing Sciences, 1(2), 215-223. | No analysis specific to a nursing activity |
| 1. Treciokiene, I., Postma, M. J., Nuygen, T., Fens, T., Petkevicius, J. et Taxis, K. (2017). A systematic review on lifestyle change interventions performed by health care professionals targeting blood pressure in hypertensive patients. Value in Health, 20 (9), A603. | Not a published systematic review |
| 1. Ulley, J., Harrop, D., Ali, A., Alton, S. et Fowler Davis, S. (2019). Deprescribing interventions and their impact on medication adherence in community-dwelling older adults with polypharmacy: a systematic review. BMC Geriatrics, 19(1), 15. | No analysis specific to a nursing activity |
| 1. van Dillen, S. M. et Hiddink, G. J. (2014). To what extent do primary care practice nurses act as case managers lifestyle counselling regarding weight management? A systematic review. BMC Family Practice, 15, 197. | Not chronic condition specific |
| 1. Weller, C. D., Buchbinder, R. et Johnston, R. V. (2016). Interventions for helping people adhere to compression treatments for venous leg ulceration. Cochrane Database of Systematic Reviews, 3, CD008378. | No analysis specific to a nursing activity |
| 1. Whiteford, C., White, S. et Stephenson, M. (2016). Effectiveness of nurse-led clinics on service delivery and clinical outcomes in adults with chronic ear, nose and throat complaints: a systematic review. JBI Database of Systematic Reviews & Implementation Reports, 14(4), 229-256. doi: 10.11124/JBISRIR-2016-2237 | Not chronic condition specific |
| 1. Wong, K. C., Wong, F. K. Y., Yeung, W. F. et Chang, K. (2018). The effect of complex interventions on supporting self-care among community-dwelling older adults: A systematic review and meta-analysis. Age and Ageing, 47(2), 185-193. | No analysis specific to a nursing activity |
| 1. Xu, H., Mou, L. et Cai, Z. (2017). A nurse-coordinated model of care versus usual care for chronic kidney disease: meta-analysis. Journal of Clinical Nursing (John Wiley & Sons, Inc.), 26(11-12), 1639-1649. doi: 10.1111/jocn.13533 | No analysis specific to primary care |
| 1. Young, K., Bunn, F., Trivedi, D. et Dickinson, A. (2011). Nutritional education for community dwelling older people: a systematic review of randomised controlled trials. International Journal of Nursing Studies, 48(6), 751-780. | No analysis specific to a nursing activity |
| 1. Chan, R. J., Marx, W., Bradford, N., Gordon, L., Bonner, A., Douglas, C., . . . Yates, P. (2018). Clinical and economic outcomes of nurse-led services in the ambulatory care setting: A systematic review. International Journal of Nursing Studies, 81, 61-80. | Not chronic condition specific |
| 1. Daly, B., Tian, C. J. L. et Scragg, R. K. R. (2017). Effect of nurse-led randomised control trials on cardiovascular risk factors and HbA1c in diabetes patients: A meta-analysis. Diabetes Res Clin Pract, 131, 187-199. doi: 10.1016/j.diabres.2017.07.019 | No analysis specific to primary care |
| 1. Shaw, R. J., McDuffie, J. R., Hendrix, C. C., Edie, A., Lindsey-Davis, L., Nagi, A., . . . Williams Jr, J. W. (2014). Effects of Nurse-Managed Protocols in the Outpatient Management of Adults With Chronic Conditions: A Systematic Review and Meta-analysis. Annals of Internal Medicine, 161(2), 113-121. doi: 10.7326/M13-2567 | No analysis specific to primary care |
| 1. Tan, S. M., Han, E., Quek, R. Y. C., Singh, S. R., Gea-Sanchez, M. et Legido-Quigley, H. (2020). A systematic review of community nursing interventions focusing on improving outcomes for individuals exhibiting risk factors of cardiovascular disease. Journal of Advanced Nursing, 76(1), 47-61. | Not chronic condition specific |
| 1. van Het Bolscher-Niehuis, M. J., den Ouden, M. E., de Vocht, H. M. et Francke, A. L. (2016). Effects of self-management support programmes on activities of daily living of older adults: A systematic review. International Journal of Nursing Studies, 61, 230-247. | No analysis specific to a nursing activity |
| 1. Weeks, G., George, J., Maclure, K. et Stewart, D. (2016). Non-medical prescribing versus medical prescribing for acute and chronic disease management in primary and secondary care. Cochrane Database of Systematic Reviews, 11, CD011227. | No analysis specific to a nursing activity |
