# Supplementary material for: Examining nursing processes in primary care settings using the Chronic Care Model: an umbrella review
Source: BMC Prim Care. 2023 Sep 4;24:176. doi: 10.1186/s12875-023-02089-3 (PMC10476383; doi:10.1186/s12875-023-02089-3)
Supplement: Supplementary file 4 — Supplementary Material 4 [file 12875_2023_2089_MOESM4_ESM.docx]

| Reviews/ AMSTAR 2 criteria | 1. Did the research questions and inclusion criteria for the review include the components of PICO? | **2. Did the report of the review contain an explicit statement that the review methods were established prior to the conduct of the review and did the report justify any significant deviations from the protocol?** | 3. Did the review authors explain their selection of the study designs for inclusion in the review? | **4. Did the review authors use a comprehensive literature search strategy?** | 5. Did the review authors perform study selection in duplicate? | 6. Did the review authors perform data extraction in duplicate? | **7. Did the review authors provide a list of excluded studies and justify the exclusions?** | 8. Did the review authors describe the included studies in adequate detail? | **9. Did the review authors use a satisfactory technique for assessing the risk of bias (RoB) in individual studies that were included in the review?** | 10. Did the review authors report on the sources of funding for the studies included in the review? | **11. If meta-analysis was performed did the review authors use appropriate methods for statistical combination of results?** | 12. If meta-analysis was performed, did the review authors assess the potential impact of RoB in individual studies on the results of the meta-analysis or other evidence synthesis? | **13. Did the review authors account for RoB in individual studies when interpreting/ discussing the results of the review?** | 14. Did the review authors provide a satisfactory explanation for, and discussion of, any heterogeneity observed in the results of the review? | **15. If they performed quantitative synthesis did the review authors carry out an adequate investigation of publication bias (small study bias) and discuss its likely impact on the results of the review?** | 16. Did the review authors report any potential sources of conflict of interest, including any funding they received for conducting the review? | Overall confidence in the results |
| --- | --- | --- | --- | --- | --- | --- | --- | --- | --- | --- | --- | --- | --- | --- | --- | --- | --- |
|  | Yes | Yes | Yes | Yes | Yes | Yes | Yes | Yes | Yes | Yes | Yes | Yes | Yes | Yes | Yes | Yes |  |
|  | No | Partial Yes | No | Partial Yes | No | No | Partial Yes | Partial Yes | Partial Yes | No | No | No | No | No | No | No |  |
|  |  | No |  | No |  |  | No | No | No |  | No meta-analysis conducted | No meta-analysis conducted |  |  | No meta-analysis conducted |  |  |
|  |  |  |  |  |  |  |  |  | Includes only NRSI |  |  |  |  |  |  |  |  |
| Backhouse et al. 2017 | Yes | Yes | No | Partial Yes | Yes | No | No | No | Yes | No | Yes | Yes | Yes | No | Yes | Yes | Moderate |
| Baker et Fatoye 2017 | Yes | Yes | No | No | Yes | No | No | Partial Yes | Yes | No | No meta-analysis | No meta-analysis conducted | Yes | No | No meta-analysis conducted | Yes | Low - Moderate |
| Caro-Bautista et al. 2020 | No | Partial Yes - Yes | No | Partial Yes | Yes | No | Yes | Partial Yes | Yes | No | Yes | Yes | Yes | Yes | Yes | Yes | High |
| Clark et al. 2010 | Yes | No | No | Partial Yes | Yes | Yes | No | Yes | Yes | No | Yes | Yes | Yes | Yes | No | Yes | High |
| Crowe et al. 2019 | Yes | No | No | Partial Yes | Yes | No | No | Partial Yes | Partial Yes | No | No meta-analysis | No meta-analysis conducted | Yes | No | No meta-analysis conducted | Yes | Low-Moderate |
| Deschodt et al. 2020 | Yes | Yes | No | Partial Yes | Yes | Yes | No | No | Yes | No | Yes | No | No | No | No | Yes | Moderate |
| Dhar et al. (2020) | Yes | No | No | No | Yes | Yes | No | No | Yes | No | No-meta-analysis | No meta-analysis conducted | No | No | No meta-analysis conducted | Yes | Critically low |
| Ekers et al. 2013 | Yes | Yes | No | Partial Yes | Yes | Yes | No | Yes | Yes | No | Yes | Yes | Yes | Yes | Yes | Yes | High |
| Facchinetti et al. 2020 | No | Yes | No | Partial Yes | Yes | Yes | No | Partial Yes | Yes | No | Yes | Yes | Yes | Yes | Yes | Yes | High |
| Gorina et al. 2018 | No | Partial Yes | No | Partial Yes | Yes | No | No | No | Yes | No | No meta-analysis | No meta-analysis conducted | Yes | No | No meta-analysis conducted | No | Moderate- high |
| Halcomb et al. 2019 | No | No | Yes | Partial Yes | Yes | No | Partial Yes | Partial Yes | Yes | No | No meta-analysis | No meta-analysis conducted | No | No | No meta-analysis conducted | Yes | Moderate |
| Han et al. 2019 | No | Yes | No | Partial Yes | Yes | Yes | No | Partial Yes | Yes | No | No meta-analysis | No meta-analysis conducted | Yes | No | No meta-analysis conducted | Yes | Moderate |
| Huntley et al. 2016 | Yes | No | Yes | Partial Yes | Yes | No | No | Partial Yes | Yes | No | Yes | Yes | Yes | No | No | Yes | Moderate |
| Latour et al. 2007 | No | No | No | Partial Yes | Yes | Yes | No | Yes | Yes | No | No meta-analysis | No meta-analysis conducted | Yes | Yes | No meta-analysis conducted | No | Moderate |
| Massimi et al. 2017 | Yes | Yes | No | Partial Yes | Yes | Yes | No | Partial Yes | Yes | No | Yes | Yes | Yes | Yes | Yes | Yes | High |
| Oeseburg et al. 2009 | No | No | No | Partial Yes | Yes | Yes | No | Partial Yes | Yes | No | No meta-analysis | No meta-analysis conducted | Yes | No | No meta-analysis conducted | No | Moderate |
| Osakwe et al. 2020 | No | No | Yes | Partial Yes | Yes | No | No | Partial Yes | Yes | No | No meta-analysis | No meta-analysis conducted | Yes | No | No meta-analysis conducted | No | Low - Moderate |
| Parker et al. 2016 | Yes | No | No | Partial Yes | No | No | Yes | Yes | Yes | No | Yes | Yes | No | Yes | Yes | Yes | Moderate |
| Rice et al. 2018 | Yes | No | Yes | Partial Yes | No | No | No | Yes | Yes | No | No meta-analysis | No meta-analysis conducted | No | No | No meta-analysis conducted | Yes | Low |
| Schadewaldt et Schultz 2011 | No | No | Yes | Partial Yes | Yes | No | No | Yes | Partial Yes | No | No | Yes | Yes | Yes | No | Yes | Low |
| Tabesh et al. 2018 | Yes | No | No | Partial Yes | Yes | Yes | No | Yes | Yes | No | Yes | Yes | No | Yes | Yes | Yes | Moderate |
| Taylor et al. 2005 | No | No | No | Partial Yes | Yes | No | Partial Yes | No | Partial Yes | No | No | No | No | No | No | Yes | Low |
| Vermeire et al. 2005 | Yes | Partial Yes | No | Yes | Yes | Yes | Yes | Partial Yes | Partial Yes | No | Yes | Yes | No | Yes | Yes | Yes | Moderate |
| Wang et al. 2019 | Yes | No | Yes | Partial Yes | No | Yes | No | Yes | Yes | No | Yes | Yes | No | No | No | No | Critically low |
| Wong et al. 2012 | Yes | Yes | No | Yes | Yes | Yes | Yes | Yes | Yes | No | Yes | Yes | Yes | No | No | No | Moderate |
| Yu-Mei Chen et al. 2019 | Yes | Partial Yes | No | Partial Yes | Yes | Yes | No | Partial Yes | No | No | Yes | No | No | Yes | No | No | Low |

**Additional File 4**

Title: Methodological assessment of included reviews using AMSTAR 2

Description: Grid for the methodological appraisal of included articles based on the 16 AMSTAR 2 criteria.
